# Inhibition of the Integrated stress response by Epstein-Barr virus oncoprotein LMP1 attenuates epithelial cell differentiation and lytic viral reactivation

**DOI:** 10.1371/journal.ppat.1012934

**Published:** 2025-02-14

**Authors:** Deo R. Singh, Yitao Zhang, Sophia J. White, Scott E. Nelson, Stuart A. Fogarty, Abigail S. Pawelski, Alisha S. Kansra, Shannon C. Kenney

**Affiliations:** 1 Department of Oncology, School of Medicine and Public Health, University of Wisconsin- Madison, Madison, Wisconsin, United States of America; 2 Department of Medicine, School of Medicine and Public Health, University of Wisconsin-Madison, Madison, Wisconsin, United States of America; University of Florida, UNITED STATES OF AMERICA

## Abstract

EBV infects normal oral keratinocytes (NOKs) and plays an essential role in undifferentiated nasopharyngeal carcinoma (NPC). We previously showed that the EBV oncogene, LMP1, promotes proliferation and inhibits spontaneous differentiation in telomerase-immortalized NOKs grown in growth factor-restricted conditions. Here we have further examined the phenotypes of NOKs infected with wild-type EBV (WT EBV) versus an LMP1-deleted EBV mutant (ΔLMP1 EBV) in growth factor-restricted conditions. RNA-seq results show that WT EBV-infected NOKs not only have reduced differentiation, but also decreased expression of genes activated by the integrated stress response (ISR) pathway, in comparison to the ΔLMP1 EBV-infected cells. The ISR pathway is mediated by increased phosphorylation of the eIF2α translation initiation factor, leading to decreased translation of most cellular proteins but increased expression of some proteins, including ATF4 and CHOP. Immunoblot analyses confirmed that WT EBV-infected NOKs have decreased phosphorylation of eIF2α in comparison to uninfected and ΔLMP1 EBV-infected cells and showed that expression of LMP1 alone is sufficient to inhibit eIF2α phosphorylation. We found that LMP1 decreases the activity of two different eIF2α kinases, PERK and GCN2, in WT EBV-infected NOKs, resulting in decreased expression of the ISR-induced transcription factors, ATF4 and CHOP, in WT EBV-infected versus uninfected and ΔLMP1 EBV-infected NOKs. Furthermore, we found that both GCN2 and PERK activity are required for efficient TPA-induced lytic EBV reactivation and TPA-mediated epithelial cell differentiation. In addition, we demonstrate that over-expression of CHOP is sufficient to induce both lytic EBV reactivation and epithelial cell differentiation in WT EBV-infected NOKs and NPC cells and show that this effect is mediated by CHOP activation of the differentiation-inducing transcription factors, KLF4 and BLIMP1. Our results suggest that inhibition of the ISR pathway by the EBV oncoprotein, LMP1, may promote early NPC development by preventing epithelial cell differentiation and lytic EBV reactivation.

## Author summary

EBV is an important cause of human malignancies, including the epithelial cell tumor, NPC. EBV+ NPCs are largely composed of latently infected cells and the latent EBV protein, LMP1, plays a key role in the development of NPC. LMP1 is a membrane protein that regulates numerous different signaling pathways, including NF-кB, PI3K and Hippo. Here we show that LMP1 inhibits the integrated stress response (ISR) pathway in normal oral keratinocytes by attenuating phosphorylation of the eIF2α translation initiation factor by the PERK and GCN2 kinases. This results in reduced expression of the ISR target proteins, ATF4 and CHOP. Importantly, we find that the ISR pathway is required for efficient epithelial cell differentiation and lytic EBV reactivation in NOKs and show that expression of CHOP alone is sufficient to induce lytic EBV reactivation and differentiation. Inhibition of the ISR pathway by LMP1 may thus promote NPC by preventing lytic EBV infection and differentiation.

## Introduction

Epstein-Barr virus (EBV) is a human gamma herpes virus that causes infectious mononucleosis and contributes to B cell and epithelial cell malignancies, including B cell lymphomas, nasopharyngeal carcinoma (NPC) and gastric cancer [1]. EBV persists in a latent form in the memory B cell population for the life of the host, while antigen-stimulated plasma cells and differentiated oropharyngeal epithelial cells support lytic viral infection [1]. The EBV-associated epithelial cell tumor, NPC, supports “type II” EBV latency, in which only three latent viral proteins (EBNA1, LMP1 and LMP2A), the small EBV-encoded nuclear RNAs (EBERs) and the viral microRNAs are expressed [2]. The mechanisms by which EBV establishes latency in NPC cells are not fully understood, although the undifferentiated phenotype of NPC tumor cells likely plays an important role in preventing the lytic form of viral infection. Lytic EBV reactivation is closely associated with epithelial cell differentiation because cellular transcription factors that are induced by, and required for, epithelial cell differentiation are used by the virus to activate its immediate-early (IE) lytic promoters [3]. Conversely, ΔNp63ɑ, a cellular transcription factor expressed at high levels in undifferentiated basal epithelial cells but not in differentiated epithelial cells reduces the activity of the BZLF1 IE gene promoter and inhibits lytic EBV reactivation [4]. Once expressed, the BZLF1 and BRLF1 EBV IE proteins collaboratively activate expression of the downstream early lytic viral proteins that mediate lytic EBV DNA replication, which is then followed by production of infectious viral particles.

The EBV latency protein LMP1 plays a key role in promoting EBV-induced B cell and epithelial cell malignancies [5]. LMP1 is a membrane protein that mimics CD40 signaling and activates multiple different downstream pathways [6]. In comparison to EBV-infected B cell lymphomas, the mechanisms by which LMP1 contributes to EBV-infected epithelial cell tumors are less well understood. Furthermore, at least some of the effects of LMP1 in EBV-infected epithelial cells may be different from its effects in EBV-infected B cells, given that LMP1 is expressed at much higher levels in EBV-transformed lymphoblastoid B-cell lines (LCLs) compared to EBV-infected epithelial cell lines [7], and EBV-infected B cells and epithelial cells have many differences in both cellular and viral gene expression.

Previous studies examining LMP1 functions in epithelial cells have often depended on over-expression of LMP1 outside the context of the intact virus, rather than comparing the phenotypes of wild-type EBV versus LMP1-deleted EBV and have also commonly been performed in carcinoma cell lines that are already fully transformed. Here we have used EBV infection of NOKs, a telomerase-immortalized normal oral keratinocyte cell line, as a more biologically relevant *in vitro* model for studying the effects of EBV infection (and LMP1 in particular) on keratinocyte phenotype. NOKs, which retain the ability to differentiate, can be stably infected (in the presence of G418 antibiotic selection) with EBV containing a G418R gene inserted into the non-essential EBV BXLF1 (TK) gene. Using the NOKs model system, we previously demonstrated that EBV infection inhibits the ability of keratinocytes to undergo differentiation in response to various types of stimuli [8] and showed that differentiation of EBV-infected NOKs induces the lytic form of EBV infection [3,7]. Furthermore, using an LMP1-deleted AG876 strain EBV mutant, we recently found that LMP1 expression is both required, and sufficient, for the ability of EBV to inhibit the spontaneous NOKs differentiation that occurs in response to growth factor deprivation in cells grown at sub-confluent density [9].

Here we have performed RNA-seq analysis to compare cellular gene expression in NOKs infected with wild-type (WT) versus LMP1-deleted (ΔLMP1) EBV when cultured in growth factor-restricted conditions. In addition to confirming our previous finding that WT EBV infection inhibits NOKs differentiation more efficiently than ΔLMP1 EBV infection, our new studies reveal that WT EBV infection prevents activation of the integrated stress response (ISR) [10] in NOKs and show that this effect is primarily mediated by LMP1. Importantly, we demonstrate that LMP1-mediated attenuation of the ISR pathway contributes to its ability to inhibit NOKs differentiation and lytic EBV reactivation and thus likely contributes to EBV-induced NPC.

## Results

### WT EBV infection, but not ΔLMP1 EBV infection, inhibits expression of ATF4 target genes in NOKs

We previously showed that NOKs infected with either AG876 strain EBV (a type 2 EBV isolated from a Burkitt lymphoma in Africa)[11], or Akata strain EBV (a type 1 EBV isolated from a Burkitt lymphoma in Japan)[12] have decreased cellular differentiation and increased proliferation in comparison to uninfected NOKs [7] and demonstrated that the EBV protein LMP1 is required for these effects [9]. To further investigate how LMP1 contributes to the phenotype of WT EBV infection in NOKs, we performed RNA-seq analysis to compare cellular gene expression in NOKs infected with WT (AG876) EBV, versus NOKs infected with an LMP1-deleted EBV mutant (ΔLMP1 EBV). The LMP1-deleted EBV mutant was constructed using CRISPR/CAS9-mediated alteration of the LMP1 gene in the AG876 EBV genome as previously described [9]. As we previously found that the effect of EBV infection on cellular proliferation and differentiation in monolayer culture is most apparent when NOKs are grown in sub-confluent conditions and in the absence of supplemental growth factors [9], the RNA-seq analysis was performed using cells grown in these conditions.

**Fig 1:**
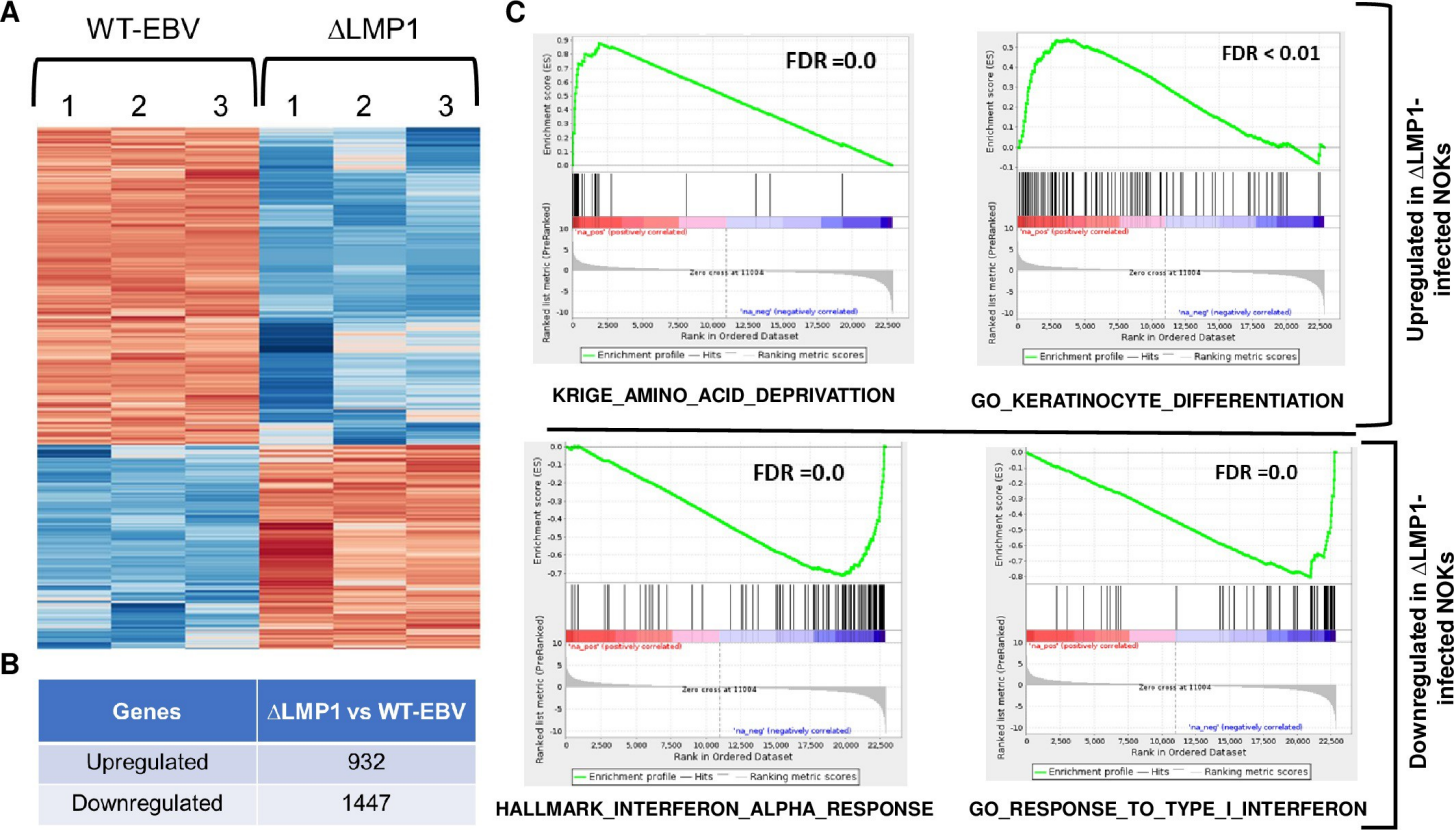
LMP1-deleted EBV-infected NOKs (ΔLMP1) have an enhanced Integrated Stress response compared to WT EBV- infected NOKs (WT-EBV). (**A**) A Heatmap comparing the top 100 differentially expressed cellular genes between NOKs infected with WT EBV versus NOKs infected with ΔLMP1 EBV is shown. The RNAs from WT-EBV and ΔLMP1 samples were harvested in triplicates when the cells were grown in growth factor-depleted media at a sub-confluent density. Red indicates a gene is upregulated in corresponding cells and blue indicates it is down-regulated. (**B**) The number of genes upregulated or downregulated in ΔLMP1 EBV-infected NOKs cells compared to WT EBV-infected NOKs is shown. (**C**) GSEA analysis showing selected gene sets or pathways up-regulated or down-regulated in ΔLMP1 EBV-infected cells compared to WT EBV-infected cells.

As shown in **Fig 1**, comparison of cellular gene expression in the ΔLMP1 EBV-infected NOKs versus the WT EBV-infected NOKs revealed 932 upregulated, and 1447 downregulated, genes in the ΔLMP1 EBV-infected NOKs. **S1 Table** compares the gene expression levels of all cellular genes in the ΔLMP1 EBV-infected versus WT EBV-infected NOKs. To identify signaling pathways regulated by LMP1 expression in EBV-infected NOKs, we analyzed the RNA-seq data using Gene Set Enrichment Analysis (GSEA). The top gene signature pattern up-regulated in the ΔLMP1 EBV-infected NOKs versus the WT EBV-infected NOKs was an amino acid depletion signature (KRIGE_AMINO_ACID_DEPRIVATION) [13] (**Fig 1C)**, likely reflecting the growth factor-restricted conditions used in this experiment. Interestingly, many of the genes differentially regulated by LMP1 in this signature are well known targets of the ATF4 transcription factor, including *DDIT3* (*CHOP*)[14], *SESN2* [15], *ASNS* [16] and *CHAC1* [17] (**Table 1**). ATF4 itself was also more highly transcribed in the ΔLMP1 EBV-infected NOKs. Another gene signature highly up-regulated in the ΔLMP1 EBV-infected NOKs versus the WT EBV-infected cells was the GO_KERATINOCYTE_DIFFERENTIATION signature (**Fig 1C**). This result confirms our previously published study showing that LMP1 inhibits differentiation in EBV-infected NOKs [9]. Two gene signatures that were highly down-regulated in the ΔLMP1 EBV-infected NOKs versus the WT EBV-infected cells were the GO_RESPONSE_TO_TYPE_I_INTERFERON signature and the HALLMARK_INTERFERON_ALPHA_RESPONSE signature (**Fig 1C**). This result is consistent with LMP1’s well known ability to increase the expression and activity of the cellular IRF7 protein [18], which activates type 1 interferon expression.

**Table 1:** ATF4 target genes activated in ΔLMP1 EBV-infected NOKs. Selected genes of interest that are upregulated in ΔLMP1 EBV-infected NOKs (ΔLMP1) compared to WT EBV-infected NOKs (WT-EBV) in the RNA-seq results are shown, along with the fold-increase in gene expression and the adjusted p value.

Gene Symbol	ΔLMP1-EBV (in FPKM)			WT-EBV (in FPKM)			log_2_Fc	Adj. p. value
	1	2	3	1	2	3		
*ATF4*	181.76	180.24	226.61	68.89	68.77	65.32	1.53	2.35E-07
*DDIT3*	14.16	19.15	24.91	2.35	2.16	2.60	3.00	2.11E-06
*SESN2*	16.96	22.96	32.05	6.11	5.26	8.18	1.86	4.59E-05
*ASNS*	4.43	4.41	4.44	0.21	0.14	0.13	4.81	1.23E-10
*CHAC1*	19.13	24.74	26.74	1.32	1.08	1.47	4.19	1.23E-10

### WT EBV infection inhibits the Integrated Stress Response (ISR) pathway via an LMP1-mediated effect

Given the finding that ATF4 target genes are more highly expressed in the ΔLMP1 EBV-infected NOKs versus the WT EBV-infected NOKs (**Table 1**), we hypothesized that LMP1 may be regulating the cellular ISR response in NOKs. The ISR is activated in response to many different types of cellular stress (including amino acid starvation and viral infection) and is mediated by four different cellular kinases, EIF2AK1 (HRI), EIF2AK2 (PKR), EIF2AK3 (PERK) and EIF2AK4 (GCN2). Each of these kinases phosphorylate the translational initiation factor eIF2α at serine 51 [10]. Although phosphorylation of eIF2α inhibits translation of most cellular proteins, it increases translation of ATF4 and leads to increased transcription and expression of ATF4 target genes [19]. ATF4-induced cellular proteins can either promote cell survival (if the stress is time-limited and reversible) or conversely induce CHOP-mediated apoptosis if the stress is irreversible [10,19].

**Fig 2:**
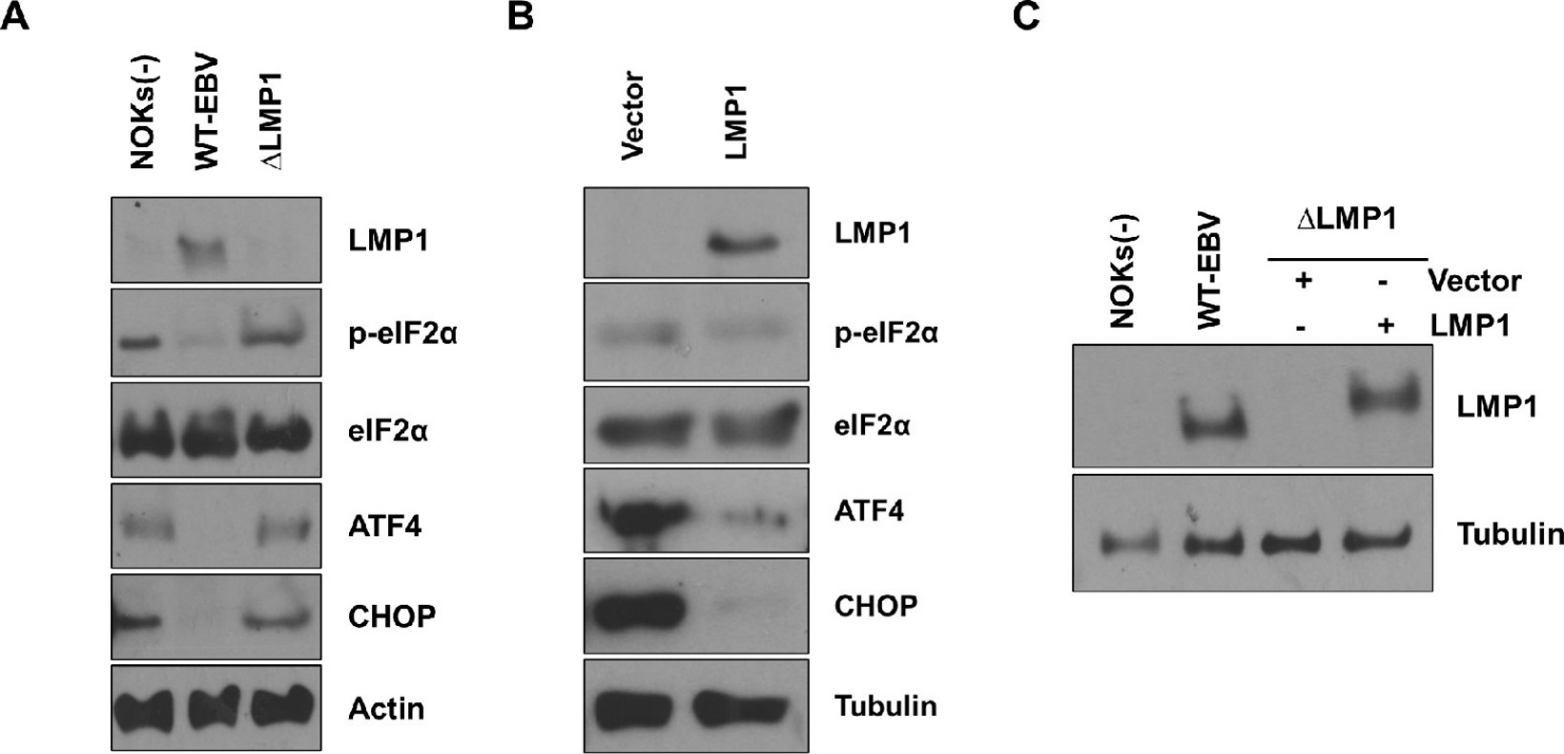
EBV inhibits the Integrated Stress Response (ISR) pathway via an LMP1-mediated effect. **(A)** Uninfected NOKs (NOKs (-)), NOKs infected with EBV (WT-EBV), or NOKs infected with ΔLMP1 EBV (ΔLMP1) were plated in the absence of growth factors (EGF and BPE) in KSFM for two days at a sub-confluent density, and then harvested to perform immunoblot analysis. Expression levels of LMP1, p-eIF2α, eIF2α, ATF4, and CHOP are shown. Actin served as a loading control. **(B)** ΔLMP1 EBV-infected NOKs infected with a LMP1-expressing lentivirus or vector control were grown in the absence of growth factors for two days at a sub-confluent density and then immunoblot analysis was performed to examine expression levels of LMP1, p-eIF2α, eIF2α, ATF4, and CHOP as shown. Tubulin served as a loading control. **(C)** Comparison of expression levels of LMP1 between WT-EBV and LMP1-overexpressing ΔLMP1 EBV-infected NOKs. Tubulin served as a loading control.

To determine if WT EBV-infected NOKs have a lower level of ISR pathway activation in comparison to ΔLMP1 EBV-infected NOKs or uninfected NOKs, cells were plated in sub-confluent conditions in the absence of any growth factors (including serum, EGF or bovine pituitary extract) for 24 hours and then immunoblot analysis was performed to compare the amount of eIF2α serine 51 phosphorylation. As shown in **Figs 2A** and **S1,** the uninfected NOKs and ΔLMP1 EBV-infected NOKs both have a higher level eIF2α S51 phosphorylation in comparison to the WT EBV-infected NOKs. Consistent with their higher level of eIF2α phosphorylation, the uninfected and ΔLMP1 EBV-infected NOKs cells also express higher levels of ATF4 and its downstream target gene, *CHOP*, in comparison to the WT EBV-infected cells. These results suggest that WT EBV infection inhibits phosphorylation of eIF2α in NOKs, resulting in decreased expression of the ATF4 and CHOP proteins, and show that LMP1 expression is required for this effect.

To confirm that LMP1 expression is sufficient to block the ISR pathway in ΔLMP1 EBV- infected NOKs, ΔLMP1 EBV-infected NOKs were stably infected with a control lentiviral vector, or a lentivirus expressing LMP1 from the type 1 EBV strain B95.8 (derived from a mononucleosis patient in the USA) [20], and the effect on eIF2α phosphorylation and other ISR pathways markers was examined. As shown in **Figs 2B** and **S2,** LMP1 expression alone is sufficient to inhibit S51 eIF2α phosphorylation, and activation of ATF4 and CHOP expression, in ΔLMP1 EBV-infected NOKs when growth factors are limiting. LMP1 expression in the LMP1 lentivirus-infected ΔLMP1 EBV-infected NOKs was similar to that in the WT EBV-infected NOKs in these experiments (**Fig 2C**). Likewise, LMP1 expression was found to be sufficient to block the ISR pathway in uninfected NOKs (**S3 Fig**). These results indicate that LMP1 expression is both required, and sufficient, for the ability of WT EBV to inhibit activation of the ISR pathway in NOKs when growth factors are restricted.

**Fig 3:**
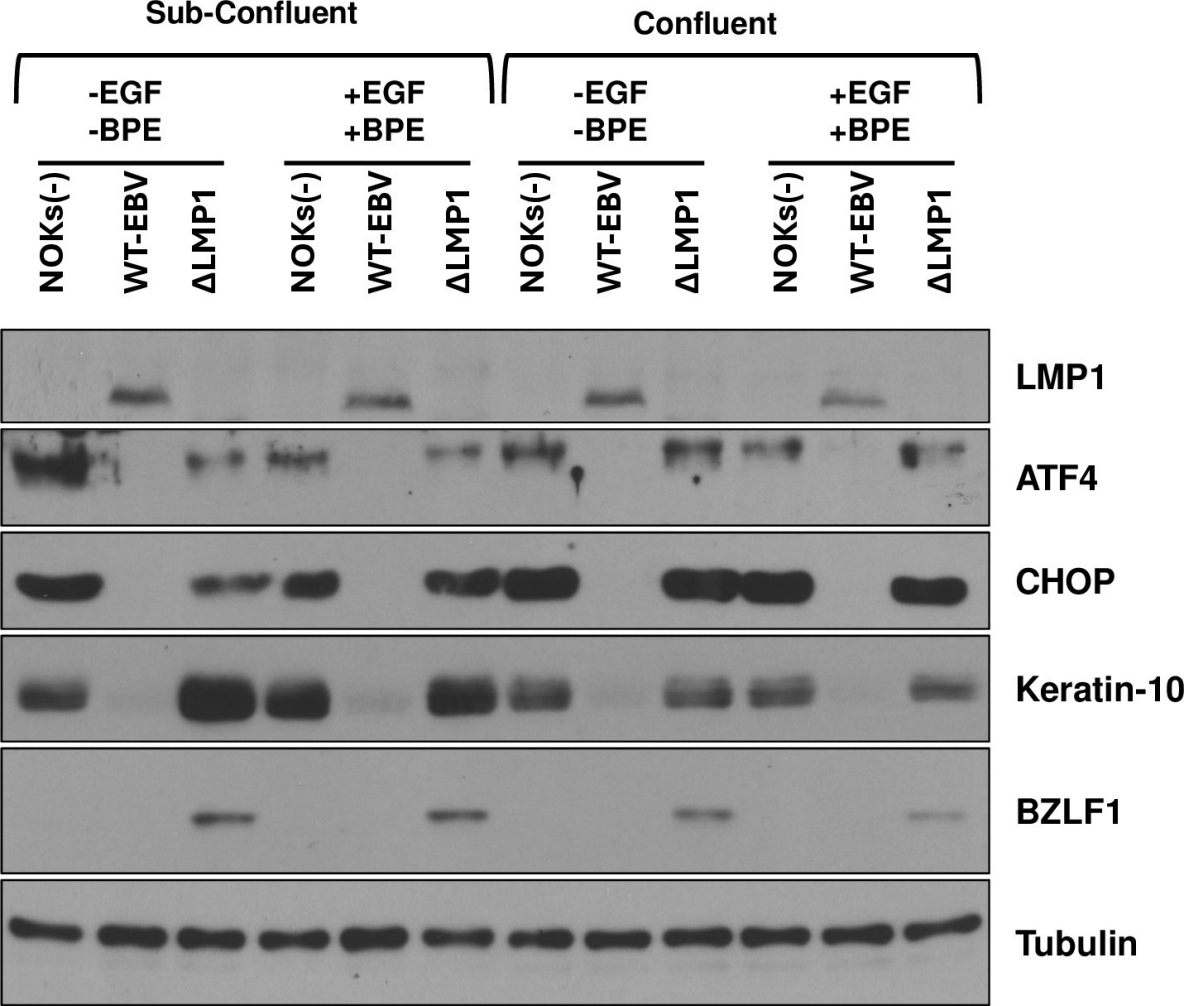
LMP1 inhibits the integrated stress response under a variety of different cell culture conditions and inhibits expression of the EBV immediate-early protein, BZLF1. Uninfected NOKs (NOKs (-)), NOKs infected with EBV (WT-EBV), or NOKs infected with ΔLMP1 EBV (ΔLMP1) were plated in the presence or absence of growth factors (EGF and BPE) in KSFM for two days at a sub-confluent or confluent density, as indicated, and then harvested to perform immunoblot analysis. Expression levels of LMP1, ATF4, CHOP, Keratin-10, and the EBV immediate-early lytic protein, BZLF1, are shown. Tubulin served as a loading control.

To determine if the ability of LMP1 to inhibit the ISR pathway in NOKs is restricted to cells cultured in growth-factor depleted conditions, and/or restricted to cells cultured in sub-confluent conditions, we also compared the levels of ATF4 and CHOP expression of uninfected NOKs, WT EBV-infected NOKs and ΔLMP1 EBV-infected NOKs cultured in the presence or absence of growth factors, or in low confluency versus high confluency conditions (**Fig 3**). These experiments revealed that WT EBV-infected cells express much less ATF4 and CHOP compared to the ΔLMP1 EBV-infected (or uninfected) NOKs even when cells were grown in the presence of growth factors and/or at high confluency. As previously noted by our group, expression of the epithelial cell differentiation protein, keratin-10, was highest in uninfected or ΔLMP1 EBV-infected NOKs cultured in growth factor-depleted, sub-confluent conditions (**Fig 3**). Furthermore, the WT EBV-infected NOKs were found to express lower levels of the immediate-early lytic protein, BZLF1, in comparison to the ΔLMP1 EBV-infected NOKs in all culture conditions tested (**Fig 3**). These results indicate that LMP1 inhibits the ISR pathway in NOKs grown in a variety of different culture conditions and show that the low level of LMP1 protein expressed in latently infected WT EBV-infected NOKs attenuates lytic EBV reactivation.

### LMP1 inhibits the activity of both the PERK (EIF2AK3) and GCN2 (EIF2AK4) kinases in NOKs

**Fig 4:**
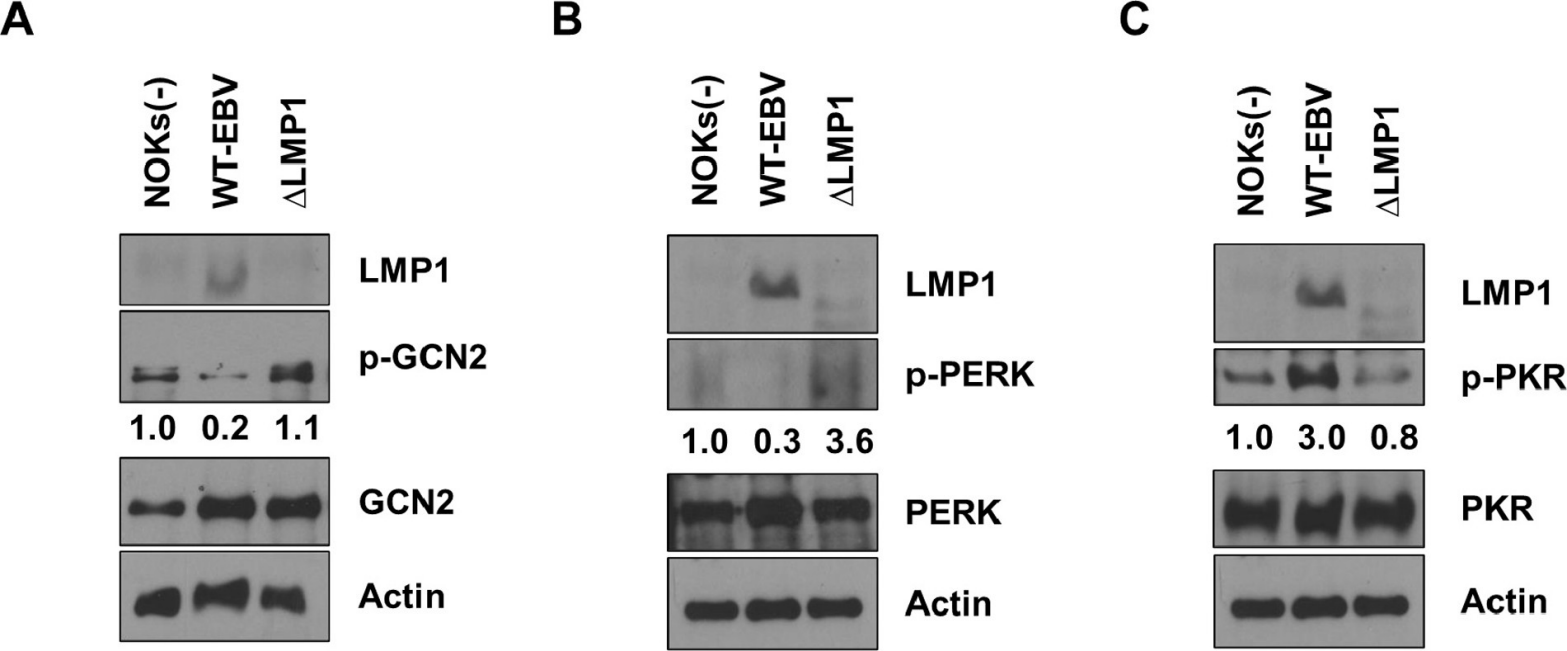
LMP1 inhibits the activity of the GCN2 and PERK kinases. Uninfected NOKs (NOKs (-)), WT EBV-infected NOKs (WT-EBV) or ΔLMP1 EBV-infected NOKs (ΔLMP1) were grown in the absence of growth factors at a sub-confluent density for two days and immunoblot analyses were performed. **(A)** Expression levels of LMP1, p-GCN2, GCN2, and Actin are shown. **(B)** Expression levels of LMP1, p-PERK, PERK, and Actin are shown. **(C)** Expression levels of LMP1, p-PKR, PKR, and Actin are shown. The levels of phosphorylated protein in each condition were quantitated using image J and normalized to the level in uninfected NOKs (set as 1). The same extracts were used in the immunoblots in (**B**) and (**C**) and probed with different antibodies. The LMP1 and Actin blots used in **(B)** and **(C)** are also same.

Although each of the four ISR kinases shares the ability to phosphorylate eIF2α and induce downstream ATF4 and CHOP expression, different cellular conditions are responsible for the activation of the individual kinases. For example, the PKR kinase is activated by double-stranded RNA (as occurs in viral infection), the PERK kinase is activated as part of the endoplasmic reticulum stress/unfolded protein response pathways, the GCN2 kinase is activated in response to amino acid depletion and the Heme Regulated Inhibitor kinase HRI (EIF2AK1) is activated by heme deficiency [19]. We hypothesized that the PKR, PERK and/or GCN2 ISR kinases would be most likely to contribute to the higher level of eIF2α phosphorylation in uninfected and ΔLMP1 EBV-infected NOKs (in comparison to the WT EBV-infected NOKs) when growth factors are limiting. To examine the relative contributions of the PKR, PERK and GCN2 ISR kinases for ATF4 and CHOP activation in uninfected, ΔLMP1 EBV-infected and WT EBV-infected NOKs, cells were grown in growth factor-depleted conditions for 24 hours and immunoblots were performed to compare the levels of PKR, PERK and GCN2 activation using phospho-specific antibodies that indicate the level of auto-activation for each kinase. WT EBV-infected NOKs were found to have a lower level of both PERK and GCN2 phosphorylation in comparison to the uninfected and ΔLMP1 EBV-infected NOKs but have a higher level of PKR phosphorylation (**Fig 4A–4C**). These results indicate that WT EBV infection inhibits the activity of both the PERK and GCN2 kinases in NOKs, although the PKR kinase is activated (consistent with the increased level of interferon signaling in these cells).

### PERK and GCN2 cooperatively activate ATF4 and CHOP expression in NOKs

**Fig 5:**
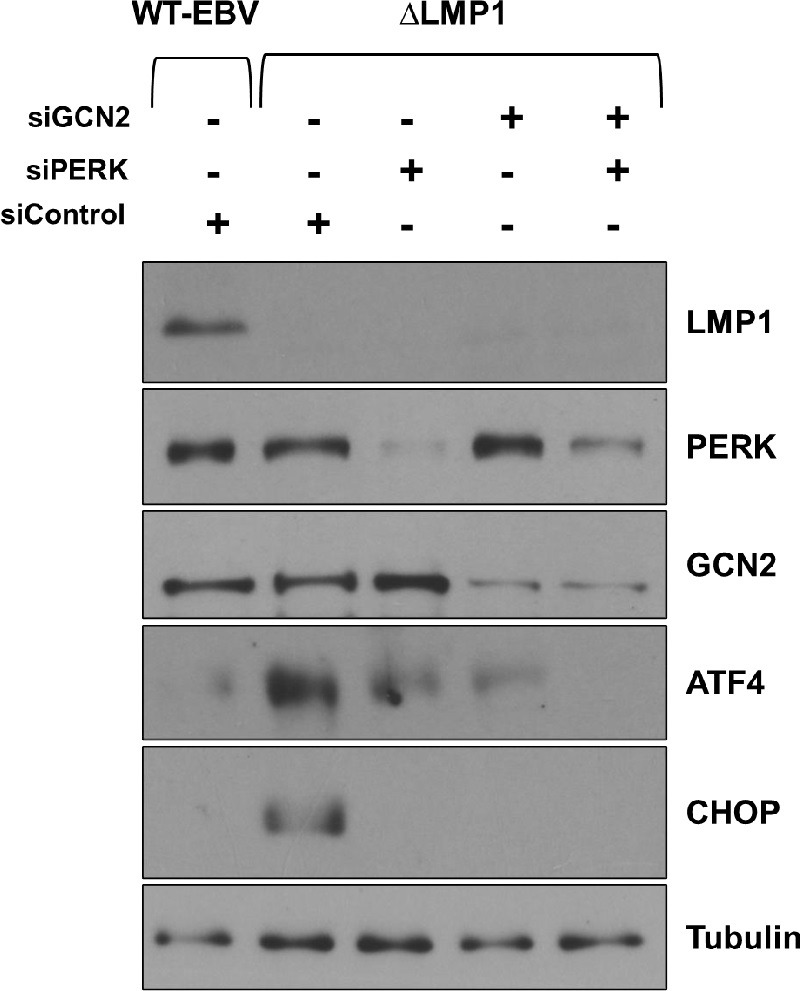
PERK and GCN2 co-operatively regulate ISR downstream targets in NOKs. WT EBV-infected NOKs (WT-EBV) or ΔLMP1-infected NOKs (ΔLMP1), grown in the absence of growth factors at sub-confluent density for two days, were transfected with control siRNA or siRNAs targeted against PERK or GCN2, alone or in combination, as indicated and immunoblots performed. Expression levels of LMP1, PERK, GCN2, ATF4, and CHOP are shown. Tubulin served as a loading control.

To determine the relative contribution of the PERK and/or GCN2 ISR kinases in activating ATF4 and CHOP expression in ΔLMP1 EBV-infected NOKs grown under growth factor-restricted conditions, cells were treated for two days with control siRNA or siRNAs directed against the PERK or GCN2 kinases (alone or in combination). As expected, the WT EBV-infected cells had less ATF4 and CHOP expression in comparison to ΔLMP1 EBV-infected NOKs in this experiment. Interestingly, individual knock-down of either the PERK or GCN2 kinases substantially blocked expression of the ATF4 protein in ΔLMP1 EBV-infected NOKs, and the combined knock-down of the PERK and GCN2 kinases together had an even more dramatic effect (**Fig 5**). Furthermore, individual knock-down of either the PERK or GCN2 kinases was sufficient to prevent CHOP expression (**Fig 5**). These results reveal that the PERK and GCN2 ISR kinases cooperatively induce ATF4 and CHOP expression in uninfected and ΔLMP1 EBV-infected NOKs grown in growth factor-restricted conditions.

### LMP1 does not activate expression of the eIF2α phosphatases, PPP1R15A and PPP1R15B

Although LMP1 was previously reported to inhibit PERK kinase activity in epithelial cells through a direct interaction with PERK [21], it has not been previously shown to inhibit the activity of the GCN2 kinase. Studies examining global protein-protein interactions between LMP1 and cellular proteins have not reported interactions between LMP1 and GCN2 [22–24]. A major autoregulatory mechanism by which the ISR pathway down-regulates itself is through ATF4-activated transcription of the PPP1R15A and PPP1R15B regulatory subunits of the PP1 phosphatase. PPP1R15A and PPP1R15B act as subunits of the PP1 phosphatase, targeting it to remove phosphorylation on the eIF2α serine 51 residue and thus terminate the ISR response [25]. To examine whether LMP1 inhibits eIF2α phosphorylation (and downstream ATF4/CHOP expression) by increasing PPP1R15A and/or PPP1R15B protein levels, we performed immunoblot analysis to compare the protein levels of each phosphatase in uninfected, WT EBV-infected and ΔLMP1 EBV-infected NOKs. As shown in **S4 Fig,** WT EBV-infected NOKs express lower levels of each of these phosphatases compared to the uninfected and ΔLMP1 EBV-infected cells, consistent with the lower levels of ATF4 in the WT EBV-infected cells. Thus, LMP1 does not regulate PERK and/or GCN2 activity by increasing the protein levels of PPP1R15A and/or PPP1R15B through an ATF4 independent mechanism.

### The GCN2 and PERK ISR kinases both contribute to TPA-induced lytic EBV reactivation and epithelial cell differentiation in EBV-infected NOKs

**Fig 6:**
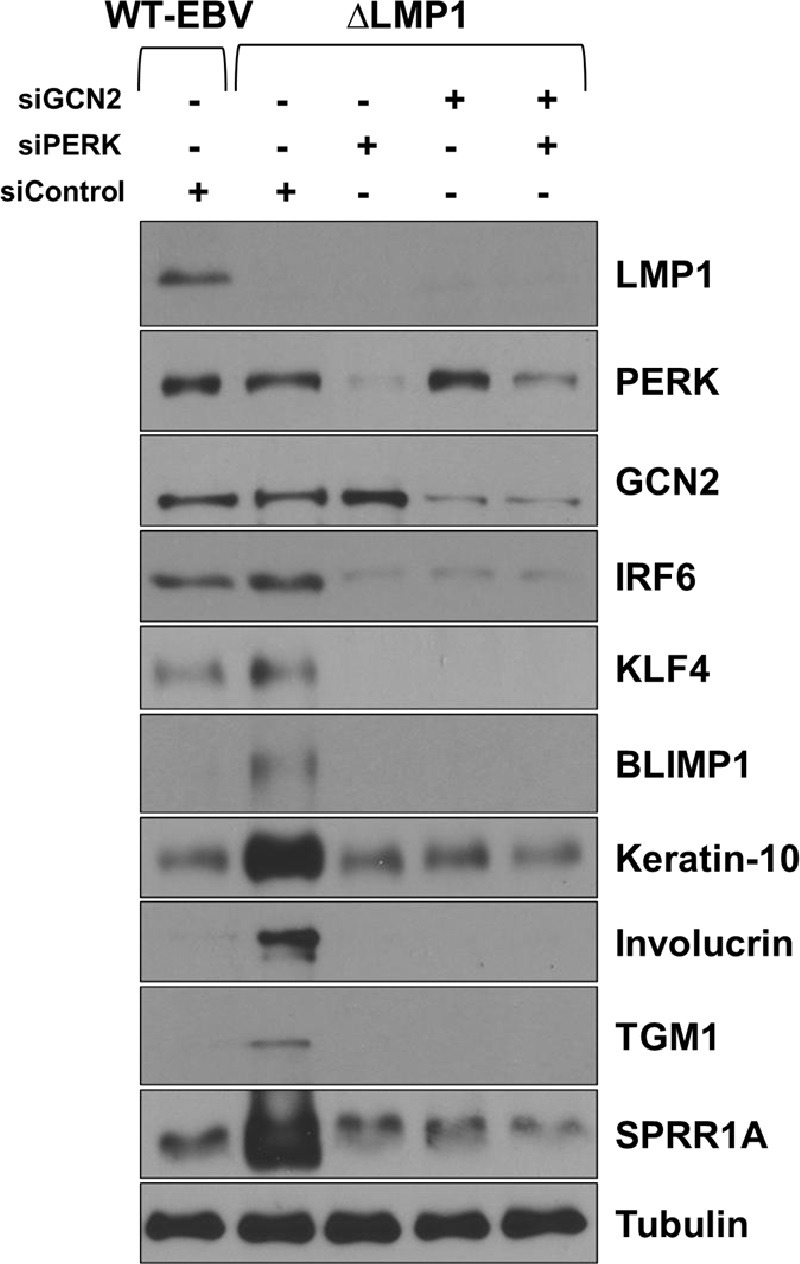
PERK and GCN2 co-operatively regulate spontaneous differentiation in NOKs. WT EBV-infected NOKs (WT-EBV) or ΔLMP1-infected NOKs (ΔLMP1), grown in the absence of growth factors at sub-confluent density for two days, were transfected with control siRNA or siRNAs targeted against PERK or GCN2, alone or in combination, as indicated and immunoblots performed. Expression levels of LMP1, PERK, GCN2, and of proteins induced by differentiation of keratinocytes (IRF6, KLF4, BLIMP1, Keratin-10, Involucrin, TGM1, and SPRR1A) are shown. Tubulin served as a loading control. The extracts used in Figure 6 immunoblots are the same as those used in Fig 5. The LMP1, PERK, and GCN2 blots are the same as those used in Fig 5.

**Fig 7:**
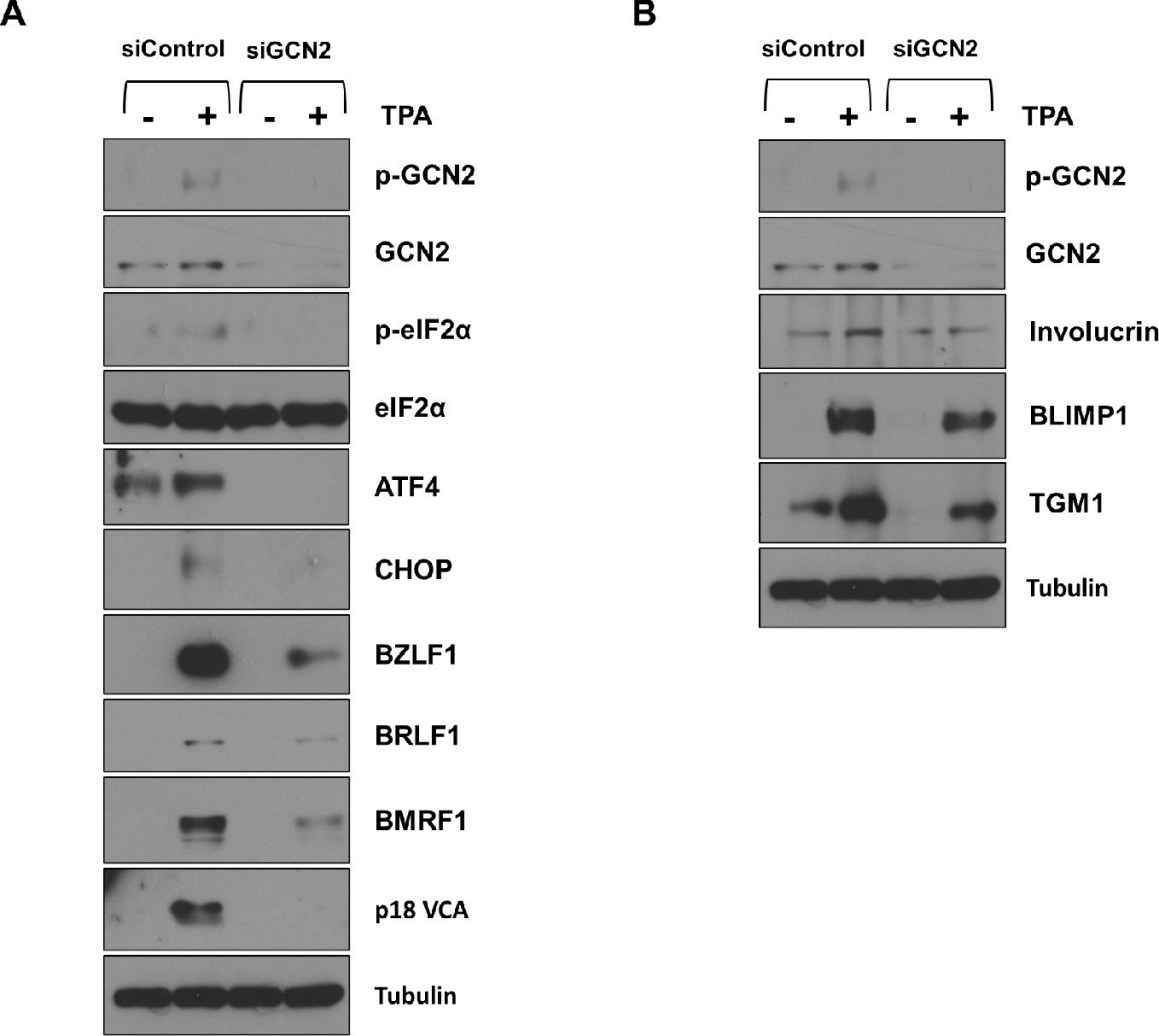
GCN2 is required for TPA-induced lytic EBV reactivation and differentiation in WT EBV-infected NOKs. WT EBV-infected NOKs cells were transfected with control siRNA or siRNAs against GCN2 as indicated, and then treated with TPA for 24 hours starting one day after siRNA transfection. **(A)** Immunoblot analysis was performed to examine the effect of GCN2 knock-down on proteins involved in the ISR pathway (including p-GCN2, GCN2, p-eIF2α, eiF2α, ATF4, and CHOP) and EBV lytic proteins (BZLF1, BRLF1, BMRF1, and p18 VCA). **(B)** Immunoblot analyses were performed to examine the effect of GCN2 knock-down on differentiation-induced cellular proteins Involucrin, BLIMP1, and TGM1. Tubulin served as a loading control for both panels. The same extracts were used for each panel, and the same GCN2, p-GCN2, and Tubulin blots were used for both panels.

**Fig 8:**
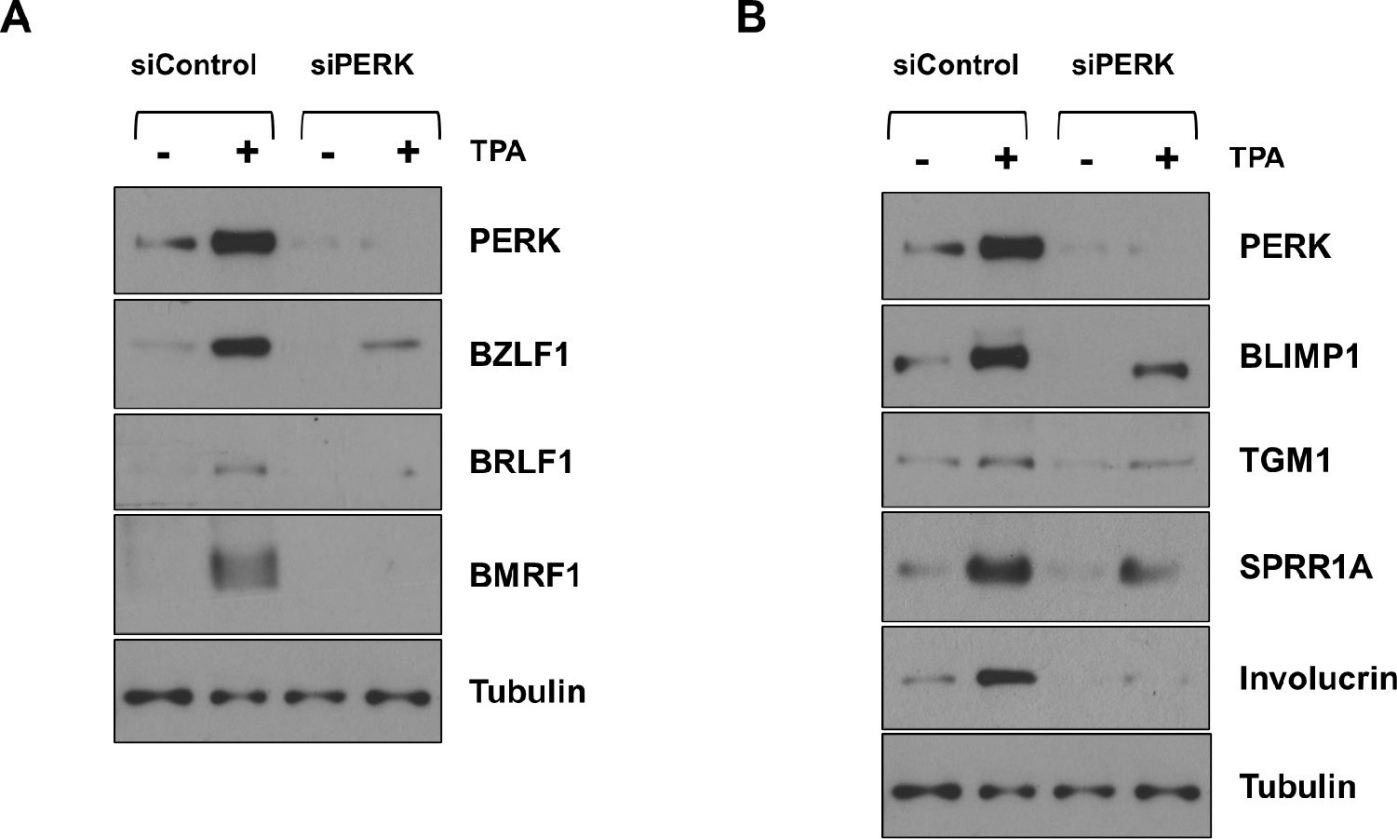
PERK is also required for efficient TPA-induced lytic EBV reactivation and differentiation in WT EBV-infected NOKs. WT EBV-infected NOKs cells were transfected with control siRNA or siRNAs against PERK as indicated, and then treated with TPA for 24 hours starting one day after siRNA transfection. **(A)** Immunoblot analyses were performed to examine the effect of PERK knock-down on EBV lytic proteins BZLF1, BRLF1, and BMRF1. **(B)** Immunoblot analyses were performed to examine the effect of PERK knock-down on differentiation-induced cellular proteins BLIMP1, TGM1, Involucrin and SPRR1A. Tubulin served as a loading control for both panels. The same extracts were used for both panels, and the same PERK and Tubulin blots were used for both panels.

Interestingly, the GCN2 ISR kinase has been shown to be both activated by, and required for, epithelial cell differentiation and certain cellular genes induced by differentiation are preferentially translated when the ISR is activated [26]. Thus, the decreased level of GCN2 activity in the WT EBV-infected NOKs might reflect the decreased level of differentiation in these cells. To determine if activation of the GCN2 and/or PERK kinases promotes lytic EBV reactivation and/or epithelial cell differentiation in NOKs, we examined the effect of knocking down GCN2 or PERK expression on spontaneous and TPA-mediated EBV lytic reactivation and differentiation in NOKs. As shown in **Fig 6,** the ability of ΔLMP1 EBV-infected NOKs to undergo spontaneous differentiation in growth factor restricted-conditions was decreased when cells were treated with either GCN2 siRNA or PERK siRNA, as indicated by the decreased levels of the differentiation-dependent cellular proteins, IRF6, KLF4, BLIMP1, Keratin-10, involucrin, TGM1 and SPRR1A. Knock-down of GCN2 in WT EBV-infected NOKs also substantially reduced TPA-induced EBV lytic reactivation (indicated by reduced expression of the immediate-early proteins BZLF1 and BRLF1, the early lytic protein BMRF1 and the late lytic protein p18 VCA) and TPA-induced epithelial cell differentiation (indicated by reduced expression of the differentiation markers involucrin, BLIMP1 and TGM1) (**Fig 7A and 7B**). Likewise, PERK knock-down also reduced TPA-mediated lytic EBV reactivation and epithelial cell differentiation in WT EBV-infected NOKs (**Fig 8A and 8B**). Furthermore, TPA treatment of EBV-infected NOKs increased both ATF4 and CHOP expression, and this effect required GCN2 **(Fig 7A**). Total PERK expression was also increased in TPA-treated cells (**Fig 8**). Thus, TPA treatment of EBV-infected NOKs activates the ISR pathway and both GCN2 and PERK activity are required for TPA-induced lytic EBV reactivation and epithelial cell differentiation.

### ATF4 and CHOP are both required for efficient TPA-induced lytic EBV reactivation and epithelial cell differentiation in WT EBV-infected NOKs

**Fig 9:**
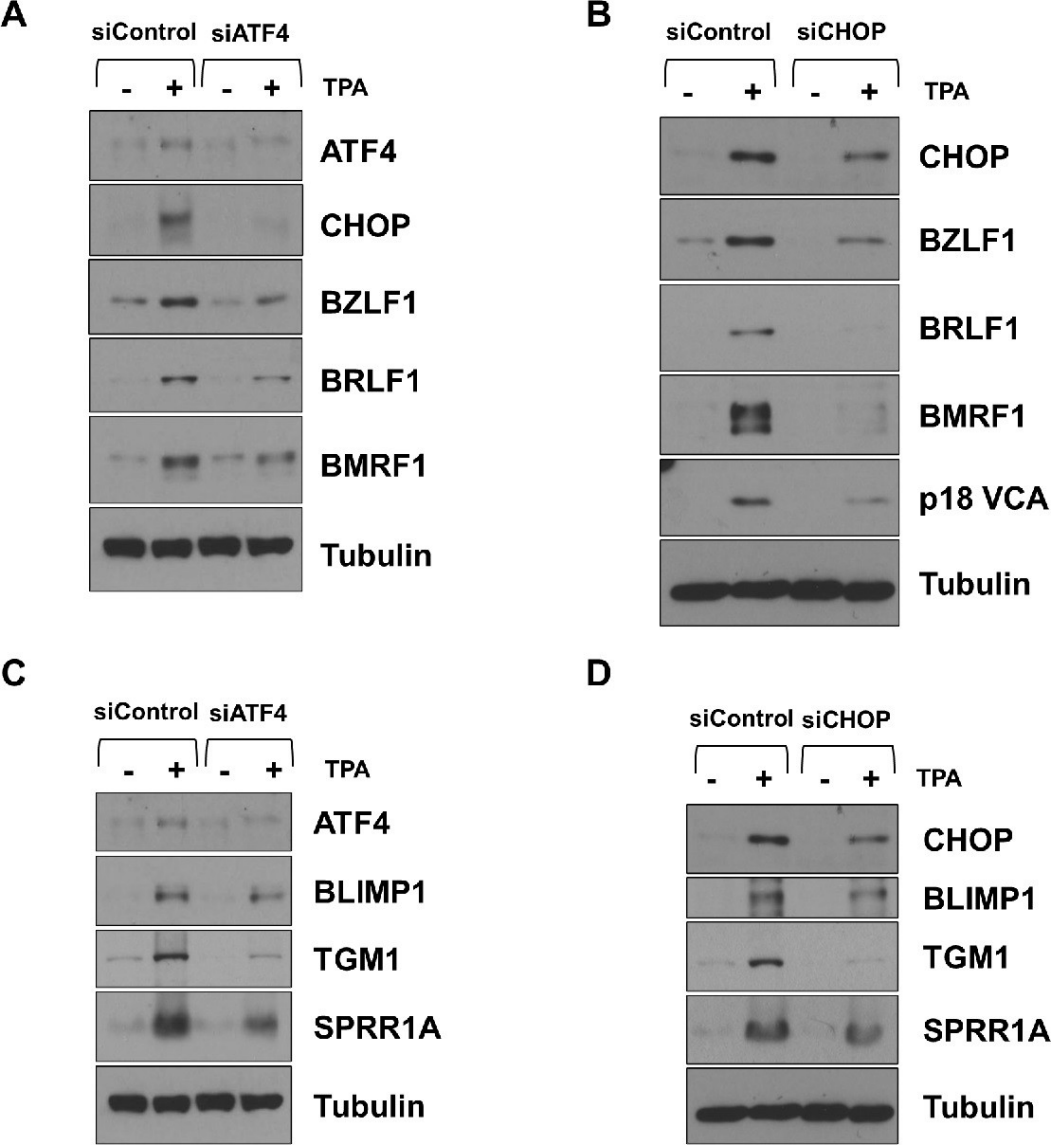
ATF4 and CHOP are required for efficient TPA-induced lytic EBV reactivation and differentiation in WT EBV-infected NOKs. WT EBV-infected NOKs cells were transfected with control siRNA or siRNAs against ATF4 **(A and C)** or CHOP **(B and D)** as indicated, and then treated with TPA for 24 hours starting one day after siRNA transfection. **(A and B)** Immunoblot analyses were performed to examine the effect of ATF4 and CHOP knock-down on lytic EBV proteins BZLF1, BRLF1, BMRF1 and p18 VCA as indicated. (**C and D)** Immunoblot analyses were performed to examine the effect of ATF4 and CHOP knock-down on TPA-induced differentiation markers BLIMP1, TGM1, and SPRR1A as indicated. Tubulin served as a loading control. The cellular extracts used in **(A)** and **(C)** or **(B)** and **(D)** were the same. The same ATF4 and Tubulin blots were used in **(A)** and **(C),** and the same CHOP and Tubulin blots were used for **(B)** and **(D)**.

Given that ISR activation regulates many different cellular gene targets by activating the ATF4 and/or CHOP transcription factors, we next compared the effects of knocking down ATF4 and CHOP expression on TPA-induced lytic EBV reactivation and/or epithelial cell differentiation in WT EBV-infected NOKs. These experiments revealed that knock-down of either ATF4 or CHOP expression inhibited TPA-induced lytic EBV reactivation and epithelial cell differentiation **(Fig 9A–9D)**. Furthermore, TPA-mediated activation of CHOP expression in WT EBV-infected NOKs was inhibited by knock-down of ATF4 (**Fig 9A**). Similar to the EBV-infected NOKs, we likewise found that knock-down of PERK, GCN2, ATF4 and CHOP each decreased the amount of spontaneous differentiation in uninfected NOKs cells (**S5 Fig**). Interestingly, knock-down of the CHOP transcription factor also resulted in decreased ATF4 expression, suggesting that CHOP auto-activates its own expression by increasing expression of ATF4. In contrast to the effect of ISR pathway inhibition on TPA-mediated lytic EBV reactivation, knock-down of various components of the ISR pathway did not inhibit the ability of EBV-infected NOKs to undergo lytic reactivation when treated with the HDAC inhibitor, SAHA **(S6 Fig)**. Together, these results indicate that the ability of TPA treatment to induce both lytic EBV reactivation and epithelial cell differentiation in WT EBV-infected NOKs is at least partially mediated by TPA-induced activation of ATF4 and CHOP.

### CHOP expression is sufficient to induce lytic EBV reactivation and epithelial cell differentiation in WT EBV-infected NOKs

**Fig 10:**
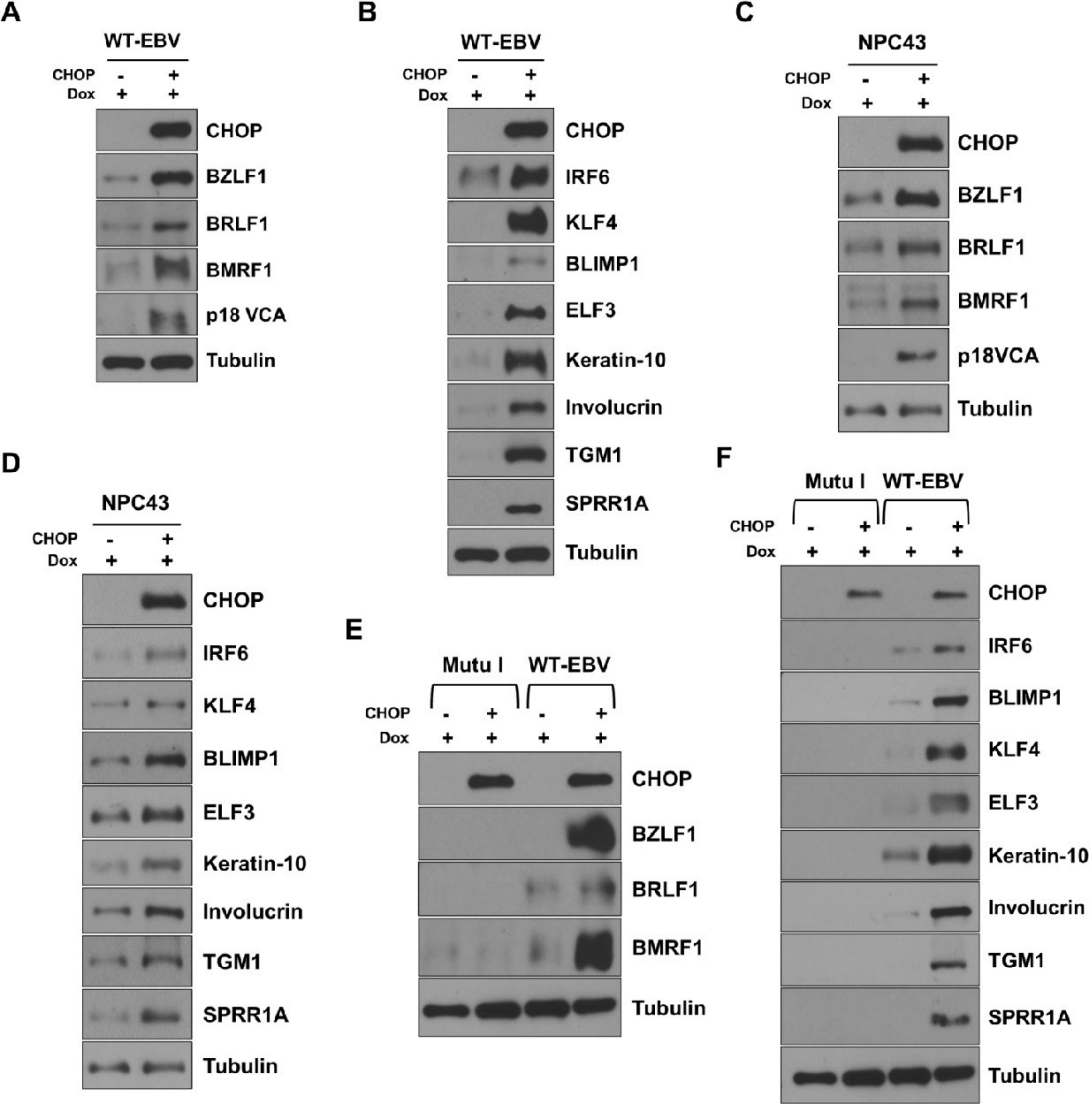
CHOP overexpression induces lytic reactivation and differentiation in NOKs and NPC43 cells but not in Mutu I Burkitt B cells. WT EBV-infected NOKs (WT-EBV), EBV infected nasopharyngeal carcinoma NPC43 cells, or EBV-infected Mutu I Burkitt lymphoma cells were stably infected with a doxycycline inducible CHOP expressing or control lentiviruses and puromycin selected to obtain stable cell lines. WT-EBV or NPC 43 cells were then treated with 500ng/ml doxycycline, and Mutu I cells were treated with 1000 ng/ml doxycycline for 48 hours and harvested to perform immunoblot analysis. (**A**) Expression of CHOP, EBV lytic markers BZLF1, BRLF1, BMRF1, p18 VCA, and Tubulin in EBV-infected NOKs. (**B**) Expression of CHOP, differentiation markers IRF6, BLIMP1, KLF4, ELF3, Keratin-10, Involucrin, TGM1, SPRR1A, and Tubulin in EBV-infected NOKs. (**C**) Expression of CHOP, EBV lytic proteins BZLF1, BRLF1, BMRF1,p18 VCA, and Tubulin in EBV- infected NPC43 cells. (**D**) Expression of CHOP, differentiation markers IRF6, BLIMP1, KLF4, ELF3, Keratin-10, Involucrin, TGM1, SPRR1A, and Tubulin in EBV-infected NPC43 cells. (**E**) Comparison of expression of EBV lytic markers BZLF1, BRLF1, and BMRF1 in EBV-infected Mutu I cells and NOKs when similar levels of CHOP expression were induced by doxycycline treatment. Tubulin serves as a loading control. (**F**) Comparison of expression of epithelial cell differentiation markers IRF6, BLIMP1, KLF4, ELF3, Keratin-10, Involucrin, TGM1, SPRR1A in EBV-infected Mutu I cells and EBV-Infected NOKs when similar levels of CHOP expression were induced. Tubulin served as a loading control.The cellular extract used to probe the blots in (**A**) and (**B**) was the same, the cellular extract used to probe the blots in (**C**) and (**D**) was the same, and the cellular extract used to probe the blots in (**E**) and (**F**) was the same. The same CHOP and Tubulin blots were used in (**A**) and (**B**), the same CHOP and Tubulin blots were used in (**C**) and (**D**), and the same Tubulin blot was used in (**E**) and (**F**).

To determine if CHOP expression alone is sufficient to induce lytic EBV reactivation in WT EBV-infected NOKs, the CHOP gene (*DDIT3*) was cloned into a lentivirus vector under the control of a doxycycline-inducible promoter as described in the Methods, and WT EBV-infected NOKs stably infected with this vector (or control vector) were selected for using puromycin. As shown in **Fig 10A** and **10B**, expression of the CHOP protein induced a high level of lytic EBV protein expression, as well as expression of epithelial cell differentiation markers, in WT EBV-infected NOKs. CHOP expression was found to induce lytic EBV reactivation and differentiation in WT EBV-infected NOKs when cells were cultured in a variety of different conditions (**S7 Fig**). Furthermore, CHOP expression was also sufficient to activate lytic EBV reactivation, and epithelial cell differentiation, in an EBV-infected nasopharyngeal carcinoma cell line (NPC43) (**Fig 10C** and **10D)**. These results confirm that activation of the ISR pathway primarily induces lytic EBV reactivation, as well as epithelial cell differentiation, via activation of CHOP expression in EBV-infected epithelial cells. Nevertheless, CHOP expression (at a similar level to that expressed in EBV-infected NOKs) did not induce lytic EBV reactivation in the EBV-positive Burkitt lymphoma lines, Mutu I (**Fig 10E**) and Raji (**S8 Fig**), and as expected did not activate expression of epithelial cell differentiation proteins such as BLIMP1 and KLF4 in B cells (**Figs 10F** and **S8)**.

### CHOP induces lytic EBV reactivation in epithelial cells via induction of differentiation-dependent transcription factors BLIMP1 and KLF4

**Fig 11:**
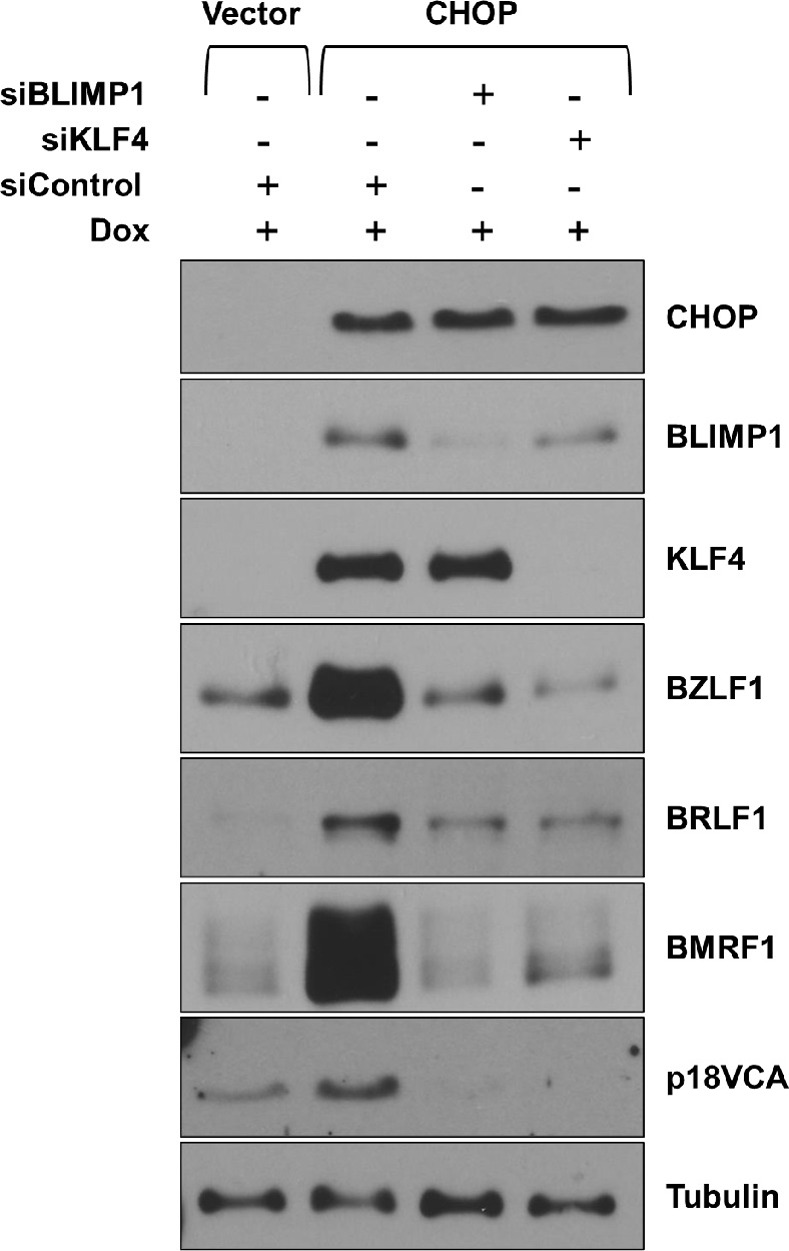
CHOP induces lytic reactivation in EBV-infected NOKs cells through transcription factors KLF4 and BLIMP1. WT EBV-infected NOKs (WT-EBV) stably infected with a doxycycline inducible CHOP-expressing or control lentivirus were transfected with control siRNA or siRNAs directed to KLF4 or BLIMP1 as indicated. 24h following transfection, cells were treated with 500ng/ml doxycycline for 48 hours and immunoblot analyses were performed to examine expression of BLIMP1, KLF4, BZLF1, BRLF1, BMRF1, and p18 VCA as shown. Tubulin served as a loading control.

As we have previously shown that lytic EBV reactivation and epithelial cell differentiation are tightly associated in WT EBV-infected NOKs, and demonstrated that epithelial cell differentiation-induced lytic EBV reactivation is at least partially mediated by the differentiation-dependent transcription factors KLF4 and BLIMP1 [3], we next asked if CHOP-mediated induction of lytic EBV reactivation in EBV-infected NOKs requires activation of the BLIMP1 and/or KLF4 transcription factors. As shown in **Fig 11,** the capacity of CHOP to induce lytic EBV reactivation in WT EBV-infected NOKs was dramatically decreased when either BLIMP1 or KLF4 was knocked down using siRNAs. These results suggest that the ability of CHOP to induce lytic EBV reactivation is epithelial cell-specific and is mediated indirectly by activation of the epithelial cell differentiation-dependent transcription factors, BLIMP1 and KLF4.

### Low level LMP1 expression decreases CHOP expression and inhibits lytic viral reactivation in NOKs, while higher level LMP1 expression (induced during differentiation) increases lytic EBV reactivation

**Fig 12:**
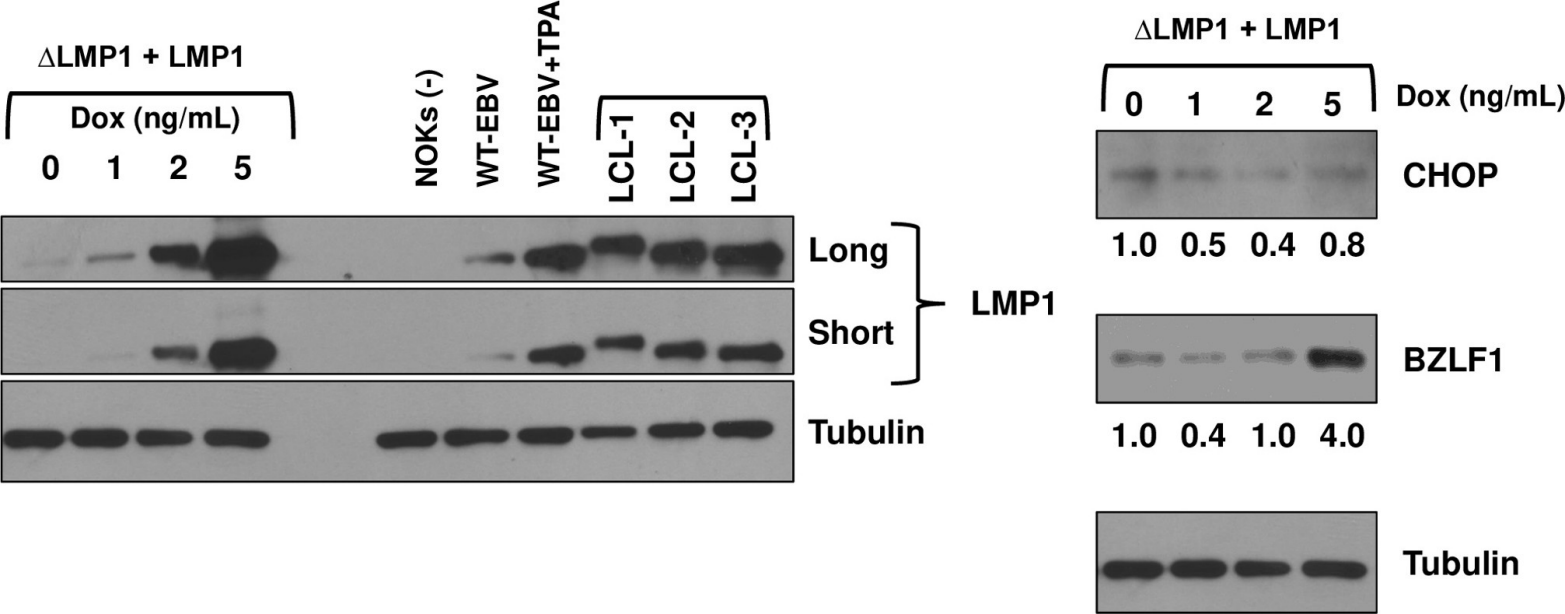
Low level and high level LMP1 expression have different effects on CHOP and BZLF1 expression in ΔLMP1 EBV-infected NOKs. ΔLMP1 EBV-infected NOKs (ΔLMP1) were stably infected with a lentivirus expressing a doxycycline inducible LMP1 gene, treated for two days with various doses of doxycycline, and then immunoblot analyses were performed to examine expression of LMP1, CHOP and BZLF1 as indicated. The levels of LMP1 expressed in untreated or TPA-treated EBV WT NOKs (WT-EBV), or in three different EBV-transformed lymphoblastoid cell lines (infected with the Mutu I (LCL-1), AG876 (LCL-2) and Akata (LCL-3) strain viruses) are shown in the left panel, and the levels of CHOP and BZLF1 are shown on the right panel. The levels of CHOP and BZLF1 were quantitated using Image J and the level in untreated cells was set as 1. Tubulin served as a loading control.

Although latently infected WT EBV-infected NOKs constitutively express LMP1 at a low level, LMP1 is also expressed in lytically infected cells at higher levels [27]. Somewhat paradoxically, although the effects of LMP1 on the ISR pathway shown here suggest that it inhibits lytic viral reactivation in NOKs by decreasing ISR-dependent cellular differentiation, we and others have previously shown that LMP1 expression contributes to lytic viral reactivation during epithelial cell differentiation [27,28]. Furthermore, we previously showed that LMP1 expression is induced by the KLF4 and BLIMP1 cellular transcription factors during epithelial cell differentiation, as well as by the BRLF1 IE protein during lytic viral reactivation [27]. We therefore hypothesized that while the low level of LMP1 expressed in latently infected NOKs inhibits viral reactivation (consistent with the results shown in **Fig 3**), the higher level of LMP1 induced by epithelial cell differentiation might promote lytic EBV reactivation. To examine this possibility, ΔLMP1 EBV-infected NOKs were stably infected with a lentivirus expressing B95.8 strain LMP1 under a doxycycline-inducible promoter, or a control lentivirus, and then cells were treated with different amounts of doxycycline to examine the effects of various levels of LMP1 expression on CHOP and BZLF1 expression. As shown in **Fig 12,** when LMP1 was expressed at a low level (similar to that expressed in latently WT EBV-infected NOKs), it decreased CHOP expression and did not induce lytic EBV reactivation. However, when LMP1 was expressed at higher levels (similar to that expressed in TPA-treated WT EBV-infected NOKs), it increased BZLF1 expression. Thus, we propose that LMP1 can either decrease, or increase, BZLF1 expression in EBV-infected NOKs, and that its effect on viral reactivation differs depending upon the level of LMP1 expression. Although our results here indicate that inhibition of the ISR pathway contributes to the ability of LMP1 to inhibit viral reactivation in latently infected cells, the mechanism(s) by which higher levels of LMP1 can enhance lytic EBV reactivation in NOKs are yet to be defined.

## Discussion

EBV infection is an important cause of human cancers, particularly B-cell lymphomas, nasopharyngeal carcinoma and gastric cancer. Although the mechanisms by which EBV transforms human B cells are relatively well understood, less is known about how EBV infection contributes to epithelial cancers such as nasopharyngeal carcinoma and gastric cancer. Given the propensity of EBV to enter the lytic form of viral infection in normal epithelial cells, resulting in death of the host cell, a key early requirement for the development of EBV-induced epithelial cell tumors must include establishing mechanism(s) by which EBV is able to maintain viral latency in these cells. In addition, the ability of EBV infection to inhibit epithelial cell differentiation also likely plays an essential role in promoting early undifferentiated NPC tumors. We recently showed that LMP1 expression in EBV-infected telomerase-immortalized normal oral keratinocyte cells (NOKs) inhibits spontaneous differentiation that occurs when growth factors are limiting [9]. Here we show that LMP1 also decreases the activity of two different ISR kinases, PERK and GCN2, in NOKs, and demonstrate that inhibition of both PERK and GCN2 activity are required for EBV’s ability to prevent epithelial cell differentiation. Furthermore, we show that both the PERK and GCN2 kinases are required for efficient lytic EBV reactivation in response to TPA-mediated differentiation. Importantly, we demonstrate that over-expression of the ISR-activated CHOP transcription factor is sufficient to induce both differentiation and lytic EBV reactivation in EBV-infected NOKs as well as in a human NPC cell line. These results are the first to reveal that activation of the integrated stress response plays an essential role in reactivating the lytic form of EBV infection in normal oral epithelial cells and NPC tumor cells and suggest that inhibition of the ISR by LMP1 promotes the development of early NPC both by inhibiting epithelial cell differentiation and preventing lytic EBV reactivation.

To our knowledge, this is the first study to show that LMP1 inhibits activity of the ISR kinase GCN2. Although the specific mechanism(s) by which LMP1 inhibits GCN2 activity in epithelial cells are not yet known, we suspect it is tied to our previous finding that LMP1 inhibits epithelial cell differentiation, given that GCN2 activity has been reported to be increased by epithelial cell differentiation [26]. Interestingly, LMP1 was previously reported to both upregulate PERK activity in EBV-infected B cells [29] and down-regulate PERK activity in EBV-infected epithelial cells [21]. LMP1 was shown to inhibit PERK activity in epithelial cells by interacting directly with PERK in the endoplasmic reticulum through a 4 amino acid motif (DLLC) in the LMP1 transmembrane domain [21]. In contrast, in EBV-infected B cells, LMP1 was shown to enhance PERK activity, although LMP1 was not found to interact directly with PERK [29], and PERK-mediated ATF4 expression in B cells was found to auto-regulate the level of LMP1. Although the reasons for these different reported LMP1 effects on PERK activity in epithelial cells versus B cells are not currently understood, the much higher level of LMP1 expression in EBV-transformed lymphoblastoid cell lines compared to EBV-infected epithelial cells [7], as well as the concomitant expression of certain EBV latency proteins (EBNA-LP, EBNA2 and the three EBNA3 proteins) in EBV-transformed LCLs that are not expressed in EBV-infected epithelial cells, may result in cell-type dependent differences. Our results here confirm the previous finding that LMP1 expressed at biologically relevant levels inhibits PERK activity in epithelial cells. In addition, our studies are the first to discover that LMP1-mediated inhibition of PERK activity, in conjunction with LMP1 inhibition of GCN2 activity, decreases both epithelial cell differentiation and lytic EBV reactivation in EBV-infected epithelial cells and thus may play an early role in promoting the development of EBV-induced NPC tumors.

Of note, strain-dependent variations in the LMP1 protein sequence have previously been proposed to alter certain LMP1 functions [30, 31]. For example, the EBV B95.8 strain LMP1 protein (isolated from a patient with infectious mononucleosis in the USA) was reported to inhibit cellular differentiation of a skin squamous cell carcinoma cell line more efficiently than the Chinese NPC-derived CAO EBV strain LMP1 protein [32]. The B95.8 strain LMP1 protein has also been reported to inhibit epithelial cell proliferation, and induce cellular cytotoxicity, more efficiently than the CAO strain LMP1 protein [32,33]. Furthermore, an EBV-BACmid expressing the CAO-derived LMP1 protein was reported to more efficiently establish latent EBV infection in transfected HEK293 cells, and more efficiently be induced into the lytic form of viral replication, in comparison to a control EBV BACmid expressing the B95.8 LMP1 protein [34]. The increased cytotoxic effect of B95.8 strain LMP1 in epithelial cells was mapped to two amino acids in the transmembrane domain of LMP1: residue 85 (which is a Leu in most NPC LMP1 strains and an Ile in B95.8 LMP1) and residue 106 (which is a Tyr in most NPC strains and a Phe in B95.8 LMP1) [33]. As shown in **S9 Fig**, the AG876 EBV strain LMP1 protein contains the NPC-associated residues at both of these positions, suggesting that it may be less cytotoxic than the B95.8 strain LMP1 in epithelial cells. Interestingly, the 4 amino acid motif (DLLC) in the LMP1 transmembrane domain previously reported to interact with PERK [21] is retained in each of the LMP1 strains shown in **S9 Fig,** including B95.8, AG876, CAO, Akata (from a Japanese Burkitt lymphoma) and M81 (from a Chinese NPC), suggesting that the ability to inhibit PERK activity may be a shared feature of many different LMP1 strains. Our studies here show that both the AG876 strain LMP1 and the B95.8 strain LMP1 (expressed in a lentivirus) proteins inhibit the ISR pathway in NOKs. However, it will be important to examine the effect of NPC-derived LMP1 proteins on the ISR pathway in NOKs in future studies.

Interestingly, although this is the first study to show that LMP1 attenuates the activity of the GCN2 kinase, the EBV BPLF1 lytic viral protein (a large tegument protein that also contains ubiquitin deconjugase (DUB) activity) was recently shown to activate the integrated stress response by increasing GCN2 activity [35]. BPLF1 was found to inhibit ubiquitination of the 40 S ribosome particle that occurs upon translational stalling, thereby inhibiting degradation of the translation-stalled polypeptides and inducing activation of the GCN2 kinase. Furthermore, enhanced GCN2 activity was shown to enhance translation of the EBV EBNA1 protein during lytic infection. Consistent with our results here, BPLF1-mediated activation of the GCN2 kinase was found to increase lytic EBV virion production [35]. Thus, EBV appears to maintain tight control of GCN2 activity to regulate the latent-to-lytic switch, encoding both a protein (LMP1) that inhibits GCN2 activity and a protein (BPLF1) that activates GCN2 activity.

Our findings that endogenous CHOP is required for TPA-induced lytic EBV reactivation in NOKs, and that CHOP over-expression is sufficient to induce lytic EBV reactivation in the absence of TPA treatment, confirm that the downstream ISR target gene, *CHOP*, is the major mediator of ISR-induced lytic EBV reactivation in epithelial cells. However, ISR-induced activation of ATF4 translation is also required for lytic EBV reactivation, as knock-down of ATF4 prevented TPA-mediated CHOP activation and lytic EBV reactivation. Interestingly, while CHOP over-expression efficiently induces lytic EBV reactivation in NOKs, we found that over-expression of CHOP is not sufficient to induce lytic EBV reactivation in the Mutu I or Raji Burkitt lymphoma B cell lines. Thus, the lytic-inducing effect of CHOP appears to be cell type specific.

Somewhat paradoxically, although our results here suggest that LMP1 can inhibit lytic EBV reactivation in normal epithelial cells by inhibiting differentiation and activation of the ISR, we and others have previously reported that LMP1 also contributes to lytic EBV infection of normal nasopharyngeal and oral keratinocytes under some circumstances [27,28]. We suspected that different levels of LMP1 expression can result in LMP1 either promoting, or inhibiting, lytic reactivation in normal epithelial cells. Using an inducible LMP1-expresing lentivirus that allowed us to tightly control LMP1 expression levels, we confirmed that low levels of LMP1 expression (similar to that found in WT EBV-infected NOKs with latent EBV infection) inhibit CHOP expression and lytic EBV reactivation, while higher levels of LMP1 expression (similar to that observed in TPA-induced lytically infected WT EBV-infected NOKs) enhance expression of the EBV IE lytic EBV protein, BZLF1. Currently, the mechanism(s) by which LMP1 contributes to lytic EBV reactivation when expressed at higher levels are not well understood.

**Fig 13:**
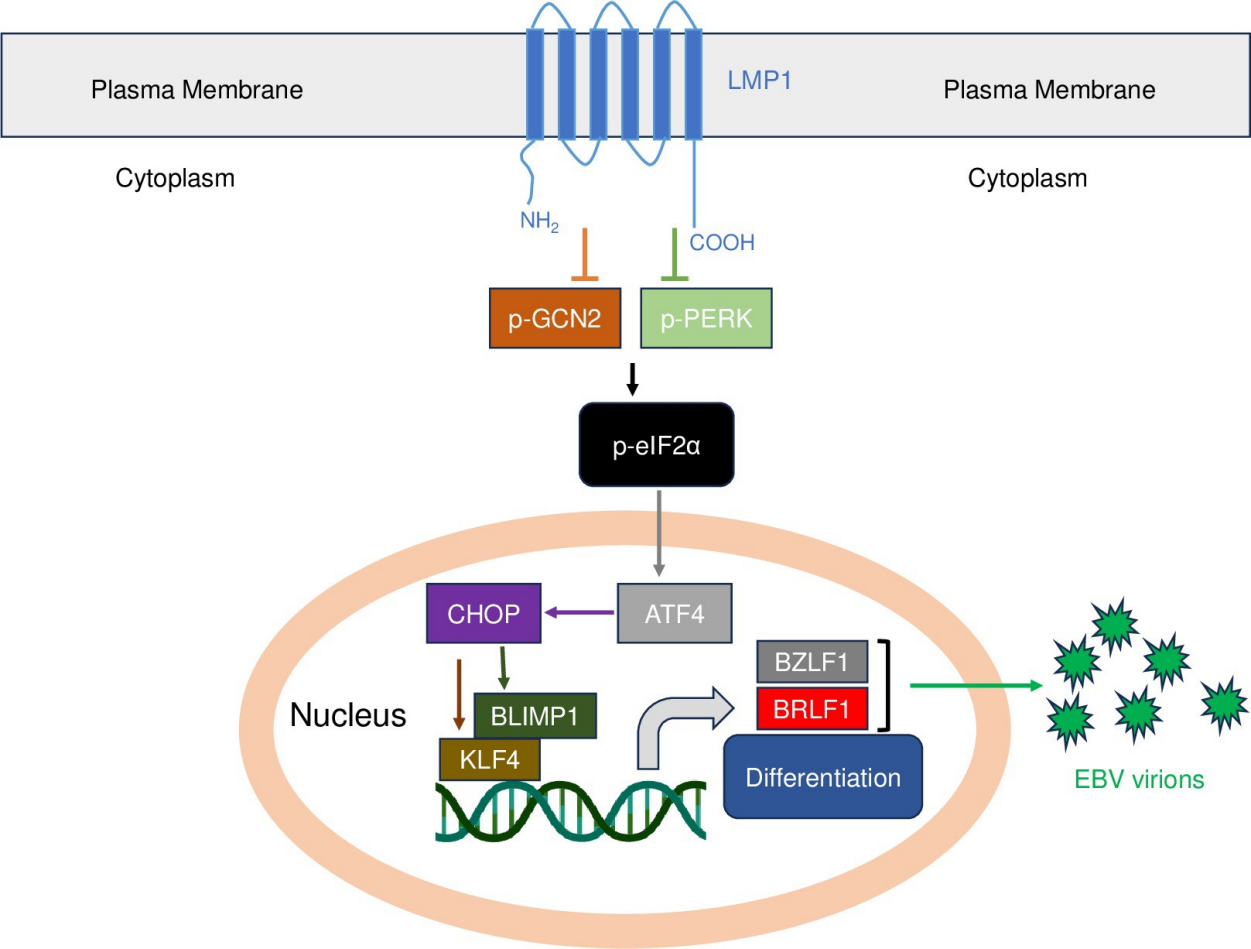
A model for how LMP1 regulates lytic EBV reactivation and epithelial cell differentiation through inhibition of the ISR pathway. See text for details of the model. The DNA symbol used in this figure was “Created with BioRender.com”.

Our results here also show that CHOP expression is required, and sufficient, for epithelial cell differentiation in NOKs. We found that CHOP induces expression of two differentiation-dependent transcription factors, BLIMP1 and KLF4, that we previously showed are sufficient to induce lytic EBV reactivation in epithelial cells. Furthermore, we demonstrated that knock-down of either KLF4 or BLIMP1 inhibited the ability of CHOP over-expression to induce lytic EBV reactivation in NOKS. Thus, our results suggest a model **(Fig 13)** in which activation of the ISR response induces lytic EBV reactivation in epithelial cells by a pathway that initially results in enhanced translation of ATF4, which then leads to increased transcription of the ATF4 target gene, *CHOP*. CHOP protein expression results in CHOP-mediated differentiation of epithelial cells and expression of the differentiation-dependent transcription factors, BLIMP1 and KLF4. BLIMP1 and KLF4 then activate expression of the EBV BZLF1 and BRLF1 immediate-early proteins, leading to lytic viral reactivation. Consistent with this model, we found that over-expression of CHOP in Mutu I and Raji Burkitt cells does not result in BLIMP1 or KLF4 expression and does not induce lytic EBV reactivation. As yet unknown are the exact mechanism(s) by which CHOP over-expression in NOKs induces epithelial cell differentiation, although it is likely to be at least partially mediated through BLIMP1 and/or KLF4 activation, given that both KLF4 and BLIMP1 promote, and are required for, epithelial cell differentiation [36,37].

In addition to EBV, other gamma herpesviruses have also been found to either regulate, and/or be regulated by, the ISR pathway. In contrast to its positive effect on lytic EBV reaction in the current study, ATF4 was found to inhibit BCR-mediated lytic gene expression in B cells infected with the MHV68 mouse gamma herpes virus by directly inhibiting the activity of the RTA immediate-early promoter, although CHOP was nevertheless found to be required for BCR-mediated MHV68 lytic replication [38]. In the case of the human gamma herpesvirus, KSHV, although lytic KSHV reactivation was shown to induce PERK activation and eIF2α phosphorylation, and activation of PERK was found to be important for efficient viral replication, the ability of PERK to induce ATF4 expression was prevented by KSHV infection and thus ATF4 and/or ATF4 target genes were not required for the lytic enhancing effect [39]. Thus, the mechanisms by which gamma herpesviruses regulate, and are regulated by, the ISR targets ATF4 and CHOP appear to be different for each virus as well as cell-type dependent in some cases.

In addition to inhibiting differentiation and lytic EBV reactivation, decreased PERK and/or GCN2 activity in LMP1-expressing epithelial cells may promote early NPC development through other mechanism(s). The roles of PERK and GCN2 in human malignancy are complex and context dependent, as PERK and GCN2 can function as either tumor suppressors or tumor drivers depending upon the tumor type and expression levels. For example, PERK mutations in human melanomas have inhibitory functions on PERK activity, and reducing PERK expression by 50% in a BRAF-dependent mouse melanoma model promoted the development of BrafV600E-induced tumors, although complete loss of PERK was tumor suppressive [40]. The GCN2 kinase can also function as a tumor suppressor. In addition to its ability to enhance epithelial cell differentiation, GCN2 was recently shown to be required for the ability of DNA damage to induce SLF911-mediated ribosomal stalling and subsequent p53-independent apoptosis [41]. Nevertheless, activation of the ISR kinases has also been shown to increase tumor metastasis by promoting epithelial-to-mesenchymal (EMT) transition and enhancing the ability of tumor cells to survive various forms of chemotherapy (reviewed in reference [42]). We suspect that while LMP1-mediated inhibition of PERK and GCN2 kinase activity may promote the earliest stages of NPC tumors, it may be deleterious for tumor survival at later stages of tumor development, helping to explain why LMP1 expression is often eventually lost in NPC tumors.

Importantly, our results here also suggest that agents that activate CHOP expression either by activating the unfolded protein response/ER stress pathway or the ISR pathway may be useful for “lytic induction” therapy of EBV+ epithelial tumors, in which the goal is to kill latently infected-EBV positive tumor cells by inducing the lytic form of viral infection. Indeed, the novel anti-cancer compound, NEO212, was recently shown to induce the lytic form of EBV infection in NPC cell lines *in vitro* via a CHOP-dependent mechanism [43]. Although we did not find that CHOP activation alone is sufficient to induce lytic EBV reactivation in the Mutu I or Raji Burkitt cell lines, a small molecule that activates the ISR kinase HRI was recently shown to induce lytic EBV reactivation in Burkitt cells [44] and clofoctol, an antibiotic that activates PERK, was likewise previously shown to induce lytic EBV reactivation in Burkitt cells through a PERK-dependent mechanism [45]. Furthermore, treatment of EBV-transformed LCLs with the ER-stress inducer, thapsigargin, also reactivated lytic EBV gene expression, although the precise mechanism(s) for this effect were not explored [46]. Given that CHOP expression alone is not sufficient to induce lytic EBV reactivation in the Mutu I or Raji Burkitt cell lines, we suspect that another ISR-induced transcription factor such as ATF4 itself or an ATF4 target gene (other than CHOP) induces lytic EBV reactivation when the ISR is activated in B cells. In any event, our results here suggest that the identification of non-toxic small molecules that specifically activate CHOP expression without activating other components of the ISR pathway might be extremely useful for developing lytic induction therapy for human EBV-positive gastric and NPC tumors.

## Materials and Methods

### Cell lines

Normal oral keratinocytes (NOKs), a gift from the Karl Munger lab at Tufts University [47], is a telomerase-immortalized normal oral keratinocyte cell line. The generation of wild-type AG876 (WT EBV)-infected NOKs (WT-EBV) and LMP1-deleted EBV-infected NOKs (ΔLMP1) was described previously [7,9]. NOKs were grown in keratinocyte serum-free media (KSFM)(GIBCO) supplemented with 12.5 mg bovine pituitary extract and 0.1 μg epidermal growth factor per 500 ml of media. 50 μg/ml of G418 antibiotic was added to the NOKs media to grow WT EBV- or LMP1-deleted EBV-infected NOKs. NPC43 (a gift from Sai Wah Tsao at the University of Hong Kong) is a nasopharyngeal carcinoma cell line described previously [48] and was grown in RPMI media supplemented with 10% fetal bovine serum, 1% pen-strep, and 4 μM ROCK inhibitor (Y-27632)(CAYMAN). The EBV infected Mutu I cell line, originally derived by the Rickinson lab at the University of Birmingham, UK [49], is a Burkitt lymphoma cell line (obtained as a gift from Jeff Sample). Raji is a Burkitt lymphoma line [50] obtained from the ATCC. Mutu I and Raji cells were maintained in RPMI media (Gibco) containing 10% fetal bovine serum (FBS), and 1% penicillin-streptomycin. HEK-293FT cells were purchased from ATCC. HEK-293FT cells were maintained in DMEM (GIBCO) supplemented with 10% FBS, 1X non-essential amino acids, and 1% penicillin-streptomycin.

### Plasmids

Plasmid DNA was prepared using the Qiagen Maxi-prep kit according to the manufacturer’s instructions. The B95.8 LMP1 gene was inserted into the lentivirus vector pCDH-MSCV-MCS-EF1a-GFP-Puro vector to obtain lentivector pCDH-MSCV-MCS-EF1a-GFP-Puro-LMP1 as previously described [9]. The plasmids pCW (Addgene Catalog number 184708, a gift from Alessia Ciarrocchi & Gloria Manzotti) was purchased from Addgene and the plasmid pCMV6-DDIT3 (Catalog Number RC201301) was purchased from Origene. To create a doxycycline-inducible CHOP vector, the *CHOP* (*DDIT3*) gene was PCR amplified from the pCMV6-DDIT3 vector using the following primers:

FW: 5’-ACCTCTAGACCACCATGGCAGCTGAGTCATTGC-3’

RV: 5’-ACCGGATCCCAAGGATGACGACGATAAGGTTTAA-3’

The PCR product was double digested with restriction enzymes XbaI and BamHI and inserted into the pCW vector at the NheI and BamHI restriction sites to obtain the pCW-CHOP vector. The cloned CHOP plasmid was sequenced by whole plasmid sequencing at Plasmidsaurus (Arcadia, CA) using Oxford Nanopore Technology with custom analysis and annotation and verified. All the primers were ordered from IDTDNA (Coralville IA).

To create a doxycycline inducible LMP1 vector, the LMP1 gene was PCR-amplified from a plasmid containing B95.8 LMP1 using the following primers:

FW: 5’-GGAGCTAGCCCACCATGGAACACGACCTTGAGAGG-3’

RV: 5’-TTAGTCATAGTAGCTTAGCTGAACTGG-3’

The PCR product purified on a Qiaquick column (Qiagen), digested with NheI (NEB) and ligated into pCW (Addgene # 184708) cut with NheI and HpaI (NEB). The sequence was confirmed by whole plasmid sequencing (Plasmidasaurus)

### Packaging of lentivectors

Lentivirus packaging was performed as previously described [3] using pCMV-VSV-G (Addgene Catalog number 8454, a gift from Bob Weinberg of the Massachusetts Institute of Technology) and psPAX2 (Addgene Catalog number 12260, a gift from Didier Trono from the École Polytechnique Fédérale de Lausanne) from Addgene. To package lentiviruses, 4 μg of the lentivirus vector, 0.6 μg of VSV-G, and 1.4 μg of psPAX2 were transfected into HEK-293FT cells plated in a 10 cm dish. Media was changed 24 hours later, and viral supernatant was harvested on days 2 and 3 to infect cells after concentrating the viral supernatant. To concentrate virus supernatant, one volume of 4 X lentivirus concentrator solution was mixed with three volumes of viral supernatant and then incubated with constant rocking at 60rpm overnight at 4°C. The solution was then centrifuged at 1600Xg for 1 hour at 4°C and the supernatant was removed without disturbing the viral pellet. The viral pellet was resuspended in 1/10^th^ the original volume of KSFM or RPMI medium and used to infect NOKs, NPC43, Mutu I or Raji cells. 4X lentivirus concentrator solution was prepared by dissolving 80g of PEG-8000 and 14g of NaCl into 80ml of MilliQ water and 20ml of 10X PBS (pH7.4), adjusting the pH to 7.2, adding water to obtain a final volume of 200ml, and filtering through a 0.2μM filter.

### Creation of EBV-infected NOKs, Mutu I or Raji cell lines stably infected with doxycycline-inducible control or CHOP-expressing lentiviral vectors

EBV-infected NOKs, Mutu I or Raji cells were infected with pCW-CHOP or control lentiviral vectors for 24 hours before selecting for lentivirus infected cells using puromycin selection (1 μg/ml). Mutu I or Raji cells were then treated with doxycyline (1000 ng/ml) while EBV-infected NOKs were treated with doxycycline (500 ng/ml) for 2 days before harvesting for western blot analysis.

### siRNAs

siRNAs against KLF4 (sc-35437), BLIMP1 (sc-37714), PERK (sc-36213), GCN2 (sc-45644), ATF4 (sc-35112), and control siRNAs A and C (sc-37007, sc-44230) were purchased from Santa Cruz. For the CHOP siRNA experiments, siRNAs against CHOP (SO-3179402G) and control siRNA (SO-3179402G) were purchased from Dharmacon. siRNAs were used at 20 pM final concentration and transfected in 6 well plates using RNAiMAX (Invitrogen) according to the manufacturer’s protocol.

### Chemicals

Phorbol 12-myristate 13-acetate (TPA) was purchased from Sigma (Catalog Number: P8139) and used at 20 ng/ml. Calyculin A, a phosphatase inhibitor, was purchased from Cell Signaling Technologies (Catalog Number: 9902) and was used to treat cells in vivo at 50nM starting 30 minutes before harvesting cells. SAHA (vorinostat) was purchased from SelleckChem (Catalog Number: S1047), dissolved in DMSO, and used at 2.5μM. Doxycycline was purchased from Sigma-Aldrich (Catalog Number: D-5207). It was dissolved in water and used at 500ng/ml, given daily. Control conditions were treated with equal amounts of the solvent.

### Cell Culture

To perform experiments at sub-confluent cell density, 250,000 cells were plated in KSFM media in the presence or absence of supplements (BPE and EGF) in each well of a 6W plate. To perform experiments at confluent density, one million cells were plated in each well of a 6W plate in KSFM media with or without supplements.

### Immunoblots

Immunoblots were performed as previously described [51]. Briefly, cell lysates were harvested with sumo lysis buffer (1:3 mixture of buffer I (5% sodium dodecyl sulfate (SDS), 0.15 M Tris-HCl (pH 6.8), 30% glycerol) and buffer II (25 mM Tris-HCl (pH 8.3), 50 mM NaCl, 0.5% NP-40, 0.5% deoxycholate, 0.1% SDS)) with protease inhibitors (cOmplete, Roche). Quantitation of protein concentration was conducted with a DC Bio-rad protein assay. The lysates were separated using a 10% polyacrylamide gel and then transferred onto a nitrocellulose membrane. The membranes were subsequently blocked with 5% milk consisting of .1% Tween 20 and 1X PBS for one hour. Membranes were then incubated with primary antibody overnight. The following day the antibodies were removed and the membrane was washed with wash buffer (1X PBS, .1% Tween 20) three times for 5 minutes. The membrane was then incubated with secondary antibody suspended in 5% milk for one hour, before washing with wash buffer three times for 10 minutes before treatment with ECL (Thermo Fisher) or Amersham ECL Prime Western Blotting Detection Reagent (Cytiva Life Sciences) and imaging.

### Antibodies

Antibodies used were the following: Anti-BRLF1 rabbit polyclonal antibody directed against the BRLF1 peptide (peptide sequence EDPDEETSSQAVKALREMAD, 1: 2500), anti-BZLF1 (Santa Cruz, catalog # sc-53904, 1:500), anti-BMRF1 (Millipore, catalog # MAB8186, 1: 2500), anti-p18 VCA rabbit polyclonal antibody directed against the p18 VCA peptide (peptide sequence GQPQDTAPRGARKKQ, 1:2000), anti-IRF6 (Sigma, catalog # HPA076162, 1:1000), anti-KLF4 (Cell Signaling Technologies, catalog # 4038, 1:1000), anti-BLIMP1 (Cell Signaling Technologies catalog # 9115S 1:1000), anti-ELF3 (Sigma, catalog #HPA003479, 1:1000), anti-Keratin-10 (Biolegend, catalog # 905404,1:10000), anti-Involucrin (Sigma, catalog #19018, 1:3000), anti-TGM1 (Novus Bio, catalog # NBP2-34062,1:10000), anti-SPRR1A (abclonal technology, catalog # A17535, 1:10000), anti-p-GCN2 (Abcam, catalog #ab75836, 1:1000), anti-GCN2 (Cell Signaling Technologies, catalog # 3302, 1:1000), anti-p-PERK (Invitrogen, catalog # MAS-15033, 1:5000), anti-PERK (Cell Signaling Technologies, catalog # 7192, 1:1000), anti-p-eIF2alpha (Cell Signaling Technologies, catalog # 9721, 1:1000), anti-eIF2alpha (Cell Signaling Technologies, catalog # 5324, 1:10000), anti-ATF4 (Cell Signaling Technologies, catalog # 11815, 1:2000), anti-CHOP (Cell Signaling Technologies, catalog #5554, 1:2000), anti-PKR (Signalway Antibody, catalog #41350, 1:5000), anti-p-PKR (Signalway Antibody, catalog #11280, 1:5000), anti-GADD34 (Protein Tech, catalog # 10449-1-AP, 1:1000), anti-PPP1R15B (Protein Tech, catalog # 14634-1-AP). The secondary antibodies used were Horseradish peroxide (HRP)- labeled goat anti-mouse antibody (Thermo Scientific# 31430, 1:5000), and HRP- labeled goat anti-rabbit antibody (Fisher Scientific 1:5000).

### Bulk RNA-seq analysis of WT EBV and ΔLMP1 EBV infected NOKs

Bulk RNA-seq libraries were prepared as previously described [52]. Briefly, AG876 wild-type (WT) EBV-infected or LMP1-deleted AG876 EBV-infected NOKs were grown in KSFM media (Thermo Fisher, Waltham, MA) without any supplements for 2 days, then harvested in TRIzol (Thermo Fisher, Waltham, MA). RNA was isolated using the Direct-zol RNA MiniPrep Kit (Zymo Research, Irvin, CA) and RNA quality was assessed using an Agilent TapeStation. A Ribo-depleted library was prepared using the TruSeq Stranded Total RNA with Ribo-Zero Plus Depletion (Illumina) library prep kit, and sequencing on an Illumina NovaSeq X with 151-bp paired-end reads was performed by the University of Wisconsin Biotechnology Center. RNA-seq analysis of host transcriptome was conducted by BioInfoRx (Madison, WI) as previously described [52]. Briefly, fastQC was used to verify raw data quality of the Illumina reads, and then reads were aligned to the GRCh38 (hg38) human genome primary assembly using Subjunc aligner from Subread [53] and assigned to genes using Ensembl annotation (v93). Raw count data was normalized for RNA composition using the TMM method [54] from EdgeR package [55], then transformed to log_2_CPM values using voom [56] method from the R Limma package [57]. A linear model was built for each comparison using R Limma package, and statistics for differential expression analysis were computed and adjusted p-values were calculated with the Benjamini-Hochberg procedure. Functional interpretation of the differentially expressed genes was conducted based on GO terms, KEGG pathway and GSEA [58,59] methods.

## Supporting information

S1 Fig EBV inhibits the Integrated Stress Response (ISR) pathway via an LMP1-mediated effect. Uninfected NOKs (NOKs (-)), WT EBV-infected NOKs (WT-EBV, clone 2), or LMP1-deleted EBV -infected NOKs (ΔLMP1, clone 2) were plated in the absence of growth factors (EGF and BPE) in KSFM for two days at a sub-confluent density, and then harvested to perform immunoblot analysis. Expression levels of LMP1, p-eIF2α, eIF2α, ATF4, and CHOP are shown. Tubulin served as a loading control. The ΔLMP1-infected clone used in this figure is a different clone than that used in Fig 2A (clone 1).
(TIF)

S2 Fig Restoration of LMP1 expression inhibits eIF2α phosphorylation and inhibits ATF4 and CHOP expression in NOKs infected with LMP1-deleted EBV. NOKs infected with LMP1-deleted EBV were infected with a LMP1-expressing lentivirus or vector control, plated in the absence of growth actors (EGF and BPE) in KSFM for two days at a sub-confluent density, and then harvested to perform immunoblot analysis. **(A)** Comparison of expression levels of LMP1 between WT-EBV and ΔLMP1 EBV-infected NOKs infected with a LMP1 lentivirus vector. **(B)** Expression levels of LMP1, p-eIF2α, eIF2α, ATF4, and CHOP are shown. Tubulin served as a loading control.
(TIF)

S3 Fig Expression of the LMP1 protein is sufficient to inhibit eIF2α phosphorylation and inhibit ATF4 and CHOP expression in uninfected NOKs. Uninfected NOKs infected with a LMP1-expressing lentivirus or vector control were plated in the absence of growth actors (EGF and BPE) in KSFM for two days at a sub-confluent density, and then harvested to perform immunoblot analysis. **(A)** Comparison of expression levels of LMP1 between WT-EBV and NOKs infected with the LMP1 lentivirus vector. **(B)** Expression levels of LMP1, p-eIF2α, eIF2α, ATF4, and CHOP are shown. Tubulin served as a loading control.
(TIF)

S4 Fig LMP1 inhibits expression of the PPP1R15A or PPP1R15B phosphatases in NOKs. Uninfected NOKs (NOKs (-)), WT EBV-infected NOKs (WT-EBV), or LMP1-deleted EBV-infected NOKs (ΔLMP1) were plated in the absence of growth factors (EGF and BPE) in KSFM for two days at a sub-confluent density, and then harvested to perform immunoblot analysis. Expression levels of LMP1, PPP1R15A (GADD34), PPP1R15B, and Tubulin are shown.
(TIF)

S5 Fig PERK, GCN2, ATF4, and CHOP expression are required for differentiation of uninfected NOKs. Uninfected NOKs were treated with control siRNA or siRNAs directed against PERK, GCN2, ATF4 or CHOP as indicated, plated in the absence of growth factors in sub-confluent conditions for two days (to induce spontaneous differentiation) and immunoblot analyses were performed to examine the expression of PERK, GCN2, ATF4 and CHOP, and the differentiation markers IRF6, TGM1, and SPRR1A. Tubulin served as a loading control.
(TIF)

S6 Fig The ISR pathway is not required for lytic EBV reactivation in response to the HDAC inhibitor, SAHA. WT EBV-infected NOKs were treated with the HDAC inhibitor, SAHA (vorinostat), or DMSO for two days in the presence or absence of siRNAs targeting PERK, GCN2 or CHOP as indicated. Immunoblot analyses were performed to examine expression of PERK, GCN2, CHOP or the early lytic EBV protein, BMRF1, as indicated. Tubulin served as a loading control.
(TIF)

S7 Fig CHOP induces lytic EBV reactivation and differentiation in WT EBV-infected NOKs grown in a variety of different culture conditions. WT EBV-infected NOKs stably infected with a doxycyline-inducible CHOP vector (+ CHOP) or a control vector (-CHOP) were plated in the presence or absence of growth factors (EGF and BPE) in KSFM for two days at a sub-confluent or confluent density, as indicated, and then treated for two days with doxycycline. Extracts were then harvested to perform immunoblot analyses to examine expression levels of CHOP, BZLF1, BMRF1, and BLIMP1 as shown. Tubulin served as a loading control.
(TIF)

S8 Fig CHOP does not induce lytic EBV Reactivation in Raji Burkitt lymphoma cells. WT EBV-infected NOKs or EBV-infected Raji Burkitt lymphoma cells were stably infected with doxycyline-inducible CHOP expressing or control lentiviruses, treated with 500ng/ml doxycycline for 48 hours, and then immunoblot analyses were performed to examine expression of CHOP, BZLF1, KLF4, and BLIMP1 as indicated. Tubulin served as a loading control.
(TIF)

S9 Fig Comparison of LMP1 sequences in different EBV strains. The LMP1 motifs previously shown to mediate PERK inhibition are shown in green and the LMP1 residues previously shown to mediate the B95.8 LMP1 epithelial cell cytotoxicity effect are shown in blue. The residues that differ from the B95.8 LMP1 protein in other EBV strains are shown in yellow.
(TIF)

S1 Table Bulk RNA-seq data comparing cellular gene expression in ΔLMP1 EBV-infected NOKs (ΔLMP1) versus WT EBV-infected NOKs (WT-EBV). (XLSX)
