# Supplementary figures and images for: Inhibition of the Integrated stress response by Epstein-Barr virus oncoprotein LMP1 attenuates epithelial cell differentiation and lytic viral reactivation
Source: PLoS Pathog. 2025 Feb 14;21(2):e1012934. doi: 10.1371/journal.ppat.1012934 (PMC11828382; doi:10.1371/journal.ppat.1012934)

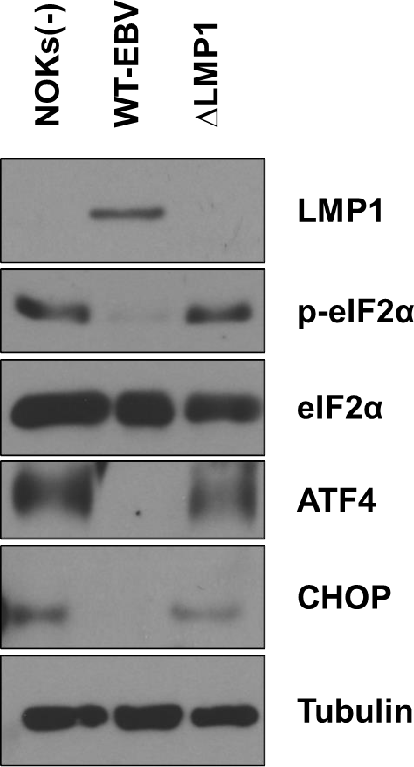

Supplement: S1 Fig — Uninfected NOKs (NOKs (-)), WT EBV-infected NOKs (WT-EBV, clone 2), or LMP1-deleted EBV -infected NOKs (ΔLMP1, clone 2) were plated in the absence of growth factors (EGF and BPE) in KSFM for two days at a sub-confluent density, and then harvested to perform immunoblot analysis. Expression levels of LMP1, p-eIF2α, eIF2α, ATF4, and CHOP are shown. Tubulin served as a loading control. The ΔLMP1-infected clone used in this figure is a different clone than that used in Fig 2A (clone 1). (TIF) [file ppat.1012934.s001.tif]

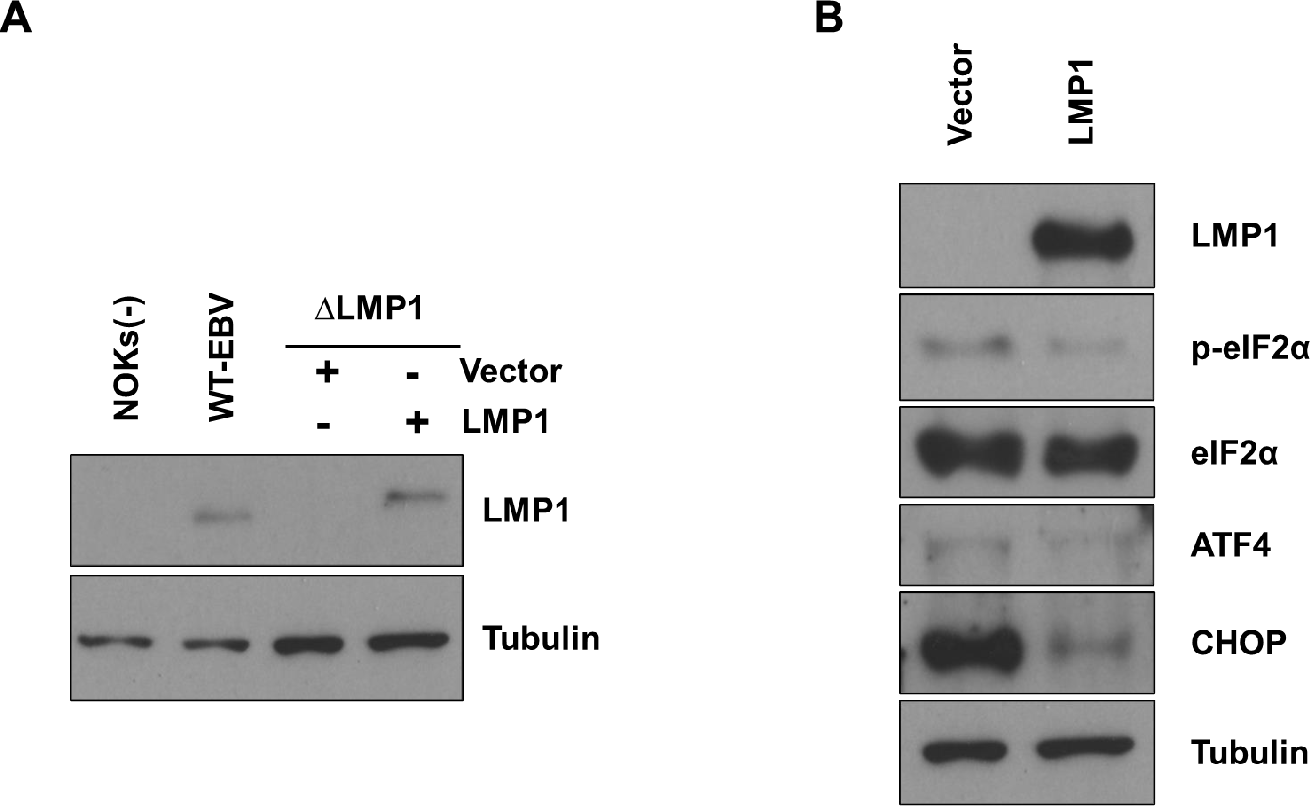

Supplement: S2 Fig — NOKs infected with LMP1-deleted EBV were infected with a LMP1-expressing lentivirus or vector control, plated in the absence of growth actors (EGF and BPE) in KSFM for two days at a sub-confluent density, and then harvested to perform immunoblot analysis. (A) Comparison of expression levels of LMP1 between WT-EBV and ΔLMP1 EBV-infected NOKs infected with a LMP1 lentivirus vector. (B) Expression levels of LMP1, p-eIF2α, eIF2α, ATF4, and CHOP are shown. Tubulin served as a loading control. (TIF) [file ppat.1012934.s002.tif]

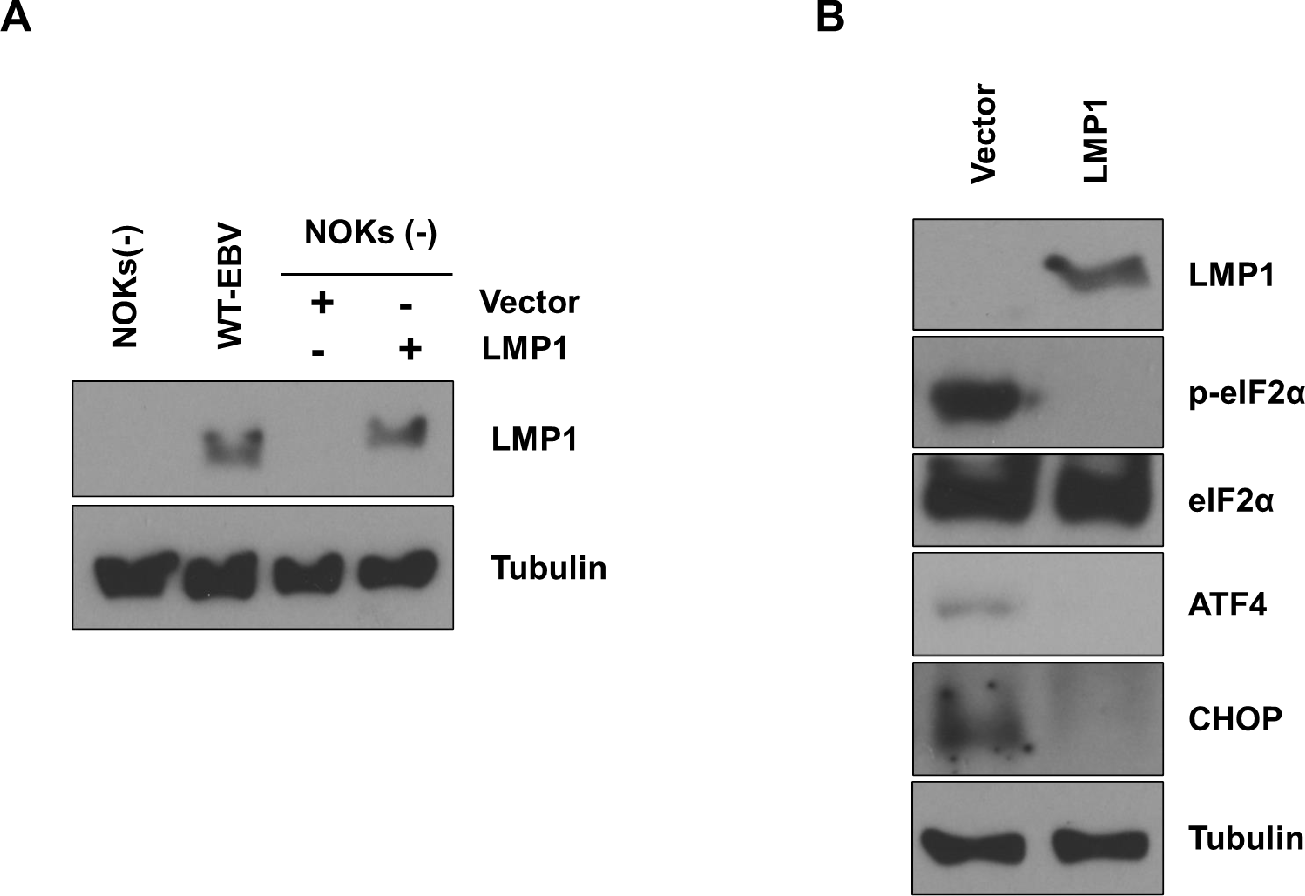

Supplement: S3 Fig — Uninfected NOKs infected with a LMP1-expressing lentivirus or vector control were plated in the absence of growth actors (EGF and BPE) in KSFM for two days at a sub-confluent density, and then harvested to perform immunoblot analysis. (A) Comparison of expression levels of LMP1 between WT-EBV and NOKs infected with the LMP1 lentivirus vector. (B) Expression levels of LMP1, p-eIF2α, eIF2α, ATF4, and CHOP are shown. Tubulin served as a loading control. (TIF) [file ppat.1012934.s003.tif]

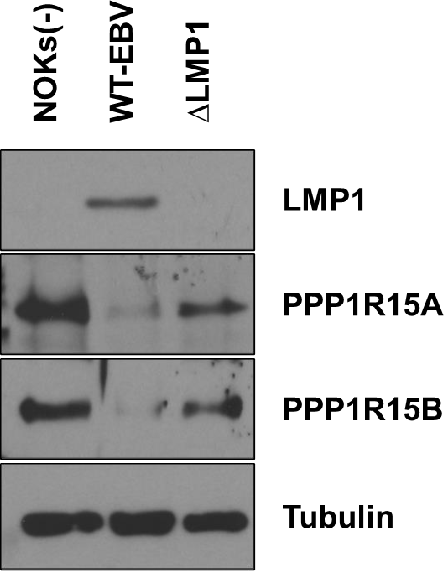

Supplement: S4 Fig — Uninfected NOKs (NOKs (-)), WT EBV-infected NOKs (WT-EBV), or LMP1-deleted EBV-infected NOKs (ΔLMP1) were plated in the absence of growth factors (EGF and BPE) in KSFM for two days at a sub-confluent density, and then harvested to perform immunoblot analysis. Expression levels of LMP1, PPP1R15A (GADD34), PPP1R15B, and Tubulin are shown. (TIF) [file ppat.1012934.s004.tif]

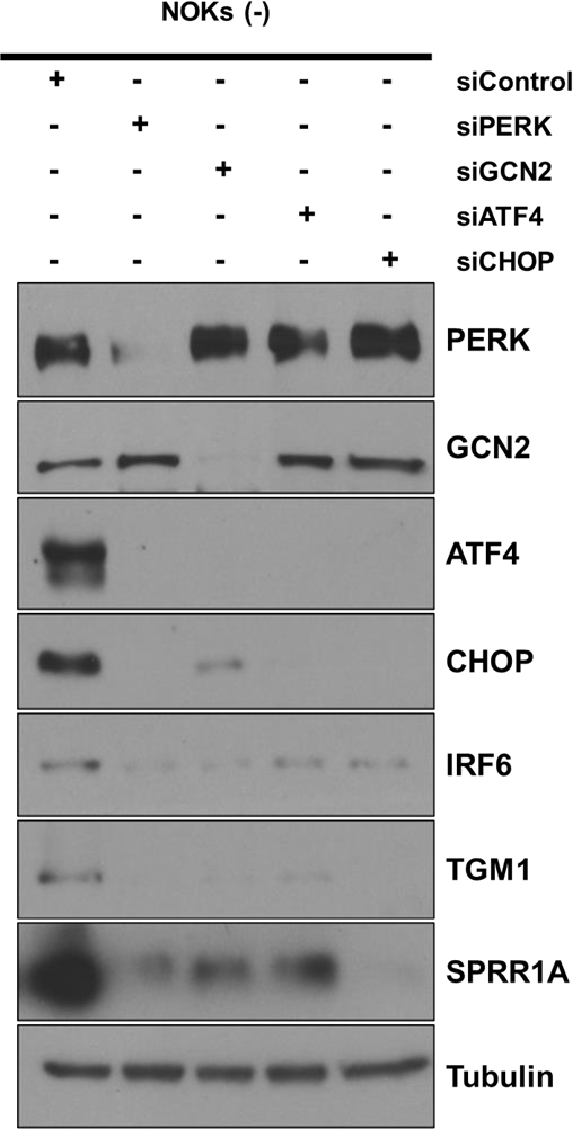

Supplement: S5 Fig — Uninfected NOKs were treated with control siRNA or siRNAs directed against PERK, GCN2, ATF4 or CHOP as indicated, plated in the absence of growth factors in sub-confluent conditions for two days (to induce spontaneous differentiation) and immunoblot analyses were performed to examine the expression of PERK, GCN2, ATF4 and CHOP, and the differentiation markers IRF6, TGM1, and SPRR1A. Tubulin served as a loading control. (TIF) [file ppat.1012934.s005.tif]

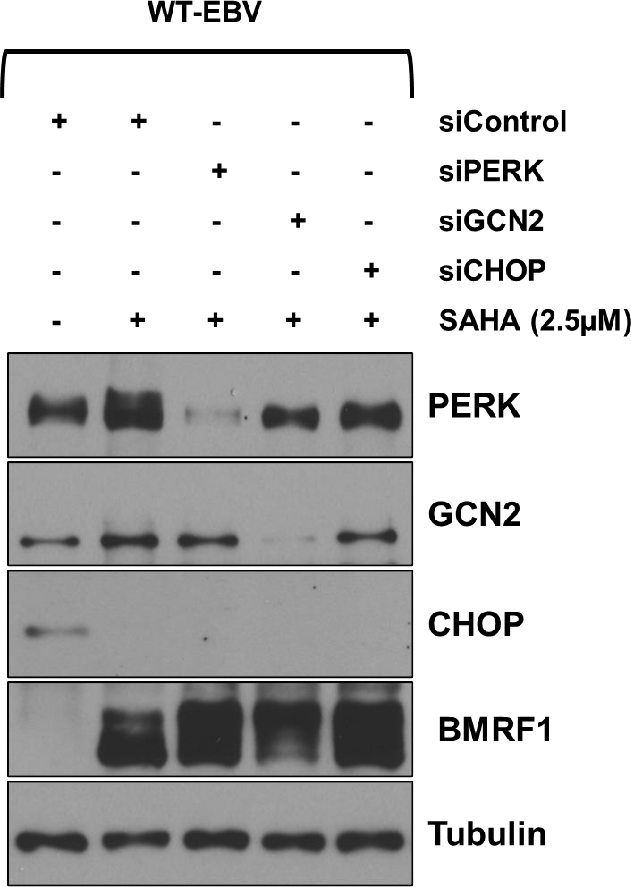

Supplement: S6 Fig — WT EBV-infected NOKs were treated with the HDAC inhibitor, SAHA (vorinostat), or DMSO for two days in the presence or absence of siRNAs targeting PERK, GCN2 or CHOP as indicated. Immunoblot analyses were performed to examine expression of PERK, GCN2, CHOP or the early lytic EBV protein, BMRF1, as indicated. Tubulin served as a loading control. (TIF) [file ppat.1012934.s006.tif]

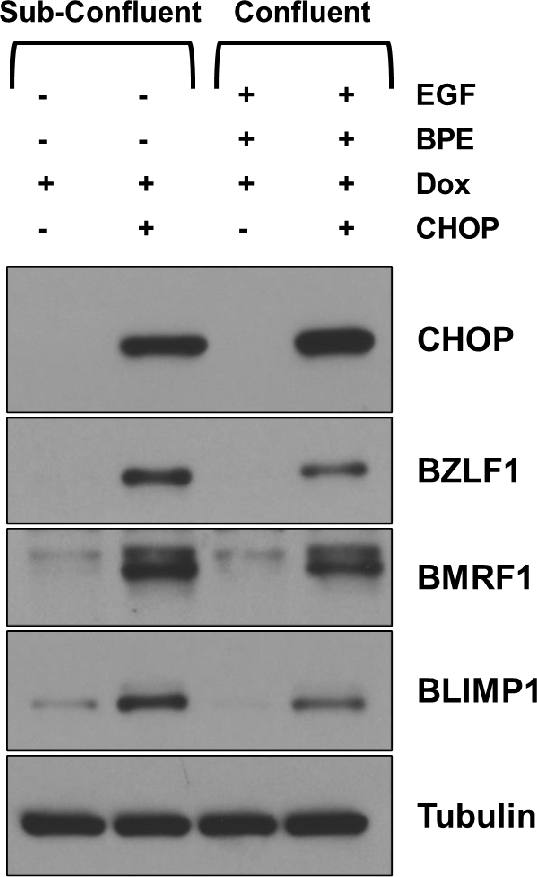

Supplement: S7 Fig — WT EBV-infected NOKs stably infected with a doxycyline-inducible CHOP vector (+ CHOP) or a control vector (-CHOP) were plated in the presence or absence of growth factors (EGF and BPE) in KSFM for two days at a sub-confluent or confluent density, as indicated, and then treated for two days with doxycycline. Extracts were then harvested to perform immunoblot analyses to examine expression levels of CHOP, BZLF1, BMRF1, and BLIMP1 as shown. Tubulin served as a loading control. (TIF) [file ppat.1012934.s007.tif]

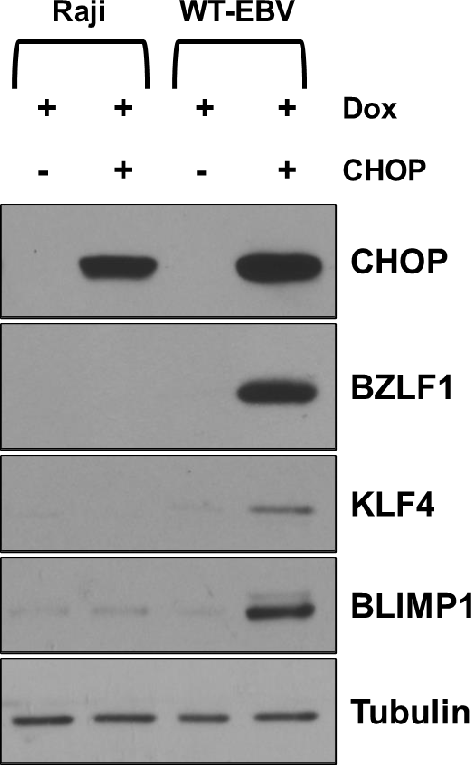

Supplement: S8 Fig — WT EBV-infected NOKs or EBV-infected Raji Burkitt lymphoma cells were stably infected with doxycyline-inducible CHOP expressing or control lentiviruses, treated with 500ng/ml doxycycline for 48 hours, and then immunoblot analyses were performed to examine expression of CHOP, BZLF1, KLF4, and BLIMP1 as indicated. Tubulin served as a loading control. (TIF) [file ppat.1012934.s008.tif]

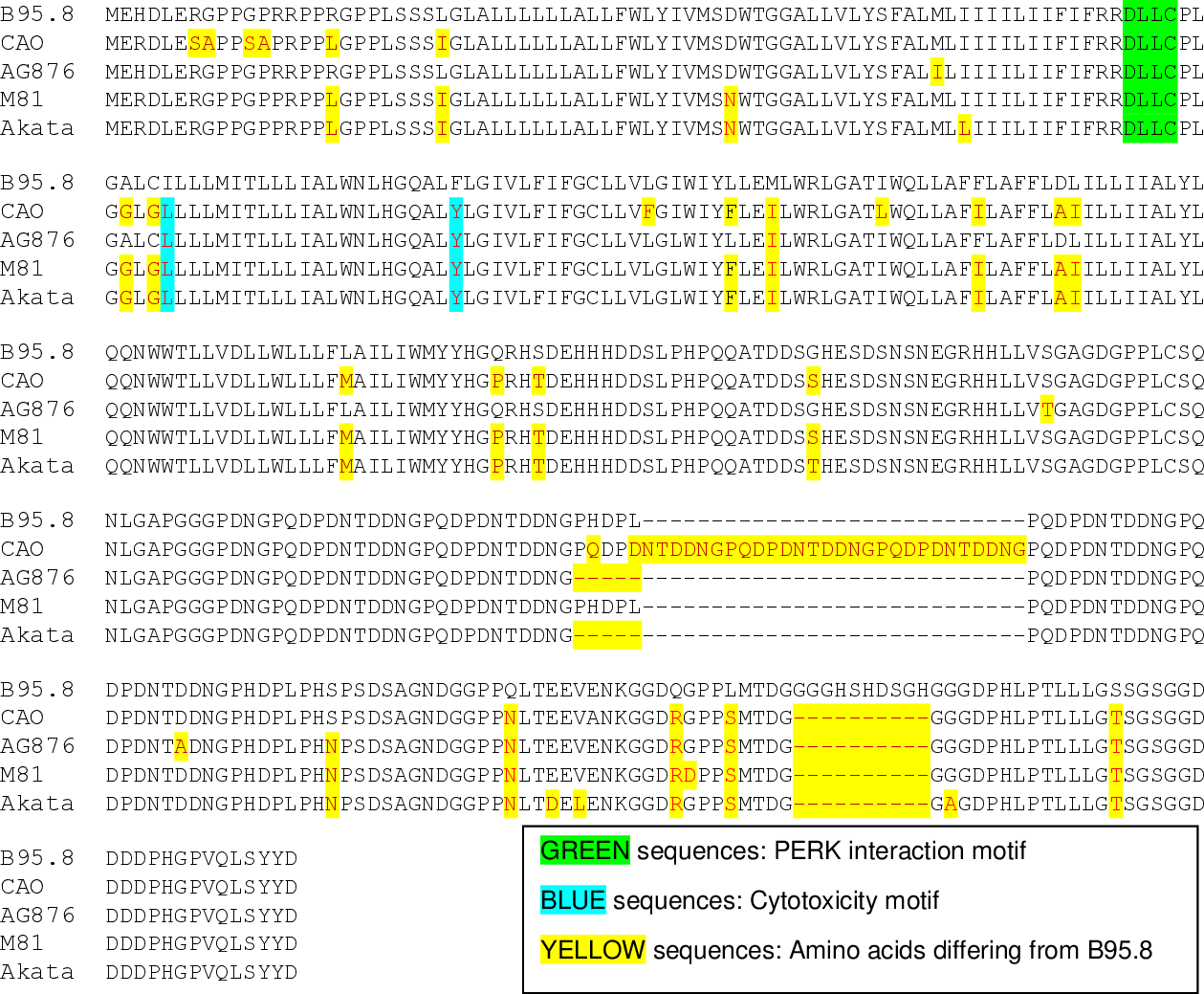

Supplement: S9 Fig — The LMP1 motifs previously shown to mediate PERK inhibition are shown in green and the LMP1 residues previously shown to mediate the B95.8 LMP1 epithelial cell cytotoxicity effect are shown in blue. The residues that differ from the B95.8 LMP1 protein in other EBV strains are shown in yellow. (TIF) [file ppat.1012934.s009.tif]
